# Synthesis and investigation on optical and electrochemical properties of 2,4-diaryl-9-chloro-5,6,7,8-tetrahydroacridines

**DOI:** 10.3762/bjoc.17.162

**Published:** 2021-09-20

**Authors:** Najeh Tka, Mohamed Adnene Hadj Ayed, Mourad Ben Braiek, Mahjoub Jabli, Peter Langer

**Affiliations:** 1 Laboratory of Asymmetric Synthesis and Molecular Engineering for Organic Electronic Materials (LR18ES19), Monastir University, Faculty of Sciences of Monastir, Environment street, 5019 Monastir, Tunisia; 2 Universität Rostock, Institut für Chemie, Albert-Einstein-Str. 3a, 18059 Rostock, Germany; 3 Department of Chemistry, College of Science Al-zulfi, Majmaah University, Al-Majmaah, 11952, Saudi Arabia,; 4 Leibniz-Institut für Katalyse e.V. an der Universität Rostock, Albert-Einstein-Str. 29a, 18059 Rostock, Germany

**Keywords:** catalysis, cross-coupling, cyclization, fluorescence, palladium

## Abstract

A facile synthesis of 2,4-diaryl-9-chloro-5,6,7,8-tetrahydroacridine derivatives is reported which is based on POCl_3_-mediated cyclodehydration followed by double Suzuki–Miyaura cross-coupling. The absorption and fluorescence properties of the obtained products were investigated and their HOMO/LUMO energy levels were estimated by cyclic voltammetry measurements. Besides, density functional theory calculations were carried out for further exploration of their electronic properties.

## Introduction

π-Conjugated organic molecules are finding use in numerous areas ranging from organic electronics [1–5], ions sensing [6–7], and solar cell development [8–10]. Within this background, acridines represent an interesting class of heteroaromatic π-conjugated compounds [11–14] which show great perspectives in medicinal chemistry [15–23], dye industries [24–26], and metal chemo sensing [27–29]. More recently, acridines have received growing attention in organic electronics [30–32], due to their strong electron-donating ability [33–34] and remarkable optoelectronic properties [32,35–37]. Acridines, as aza-analogues of anthracene have obtained much attention in the field of organic light emitting diodes [38–41].

**Figure 1:**
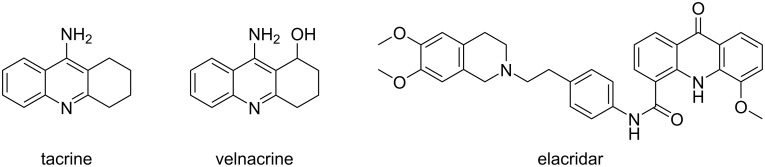
Some important tetrahydroacridines used as drugs.

While considerable attention has been devoted to the photophysical properties of acridines, not much work has been reported related to their partially hydrogenated analogues, even though they were used as electron-donor groups for OLED applications [42–43]. On the other hand, tetrahydroacridines have received much attention in medicinal chemistry, due to their ability to inhibit topoisomerase enzymes and block the DNA transcription [44–45]. In particular, they have been widely explored for the treatment of Alzheimer's disease [46–51], human cancer [52–53], and tuberculosis [54] (Figure 1).

Taking into consideration the significant medicinal potential of tetrahydroacridines and the lack of knowledge concerning their optoelectronic properties, the search for new candidates is of ongoing interest. Recently, considerable attention has been given to acridines in our laboratory and we developed new synthetic methods for dibenzoacridines and acridones based on Pd-catalyzed cross-coupling reactions [55–56]. More recently, we have reported the synthesis of alkynylated 5,6,7,8-tetrahydroacridines [57]. In continuation to our previous work and as a part of our interest in new organic materials [58–60], we report herein the synthesis of hitherto unknown 2,4-diaryl-9-chloro-5,6,7,8-tetrahydroacridine derivatives and their optical and electrochemical properties investigation by the use of UV–vis and emission spectroscopy, CV measurements as well as DFT calculations.

## Results and Discussion

**Scheme 1:**
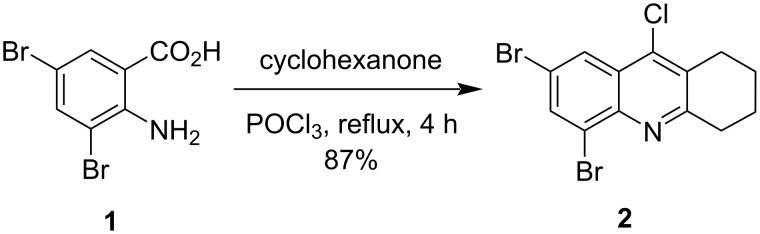
Synthesis of **2**.

**Synthesis.** At the beginning of this study, we synthesized 2,4-dibromo-9-chloro-5,6,7,8-tetrahydroacridine (**2**) by refluxing 3,5-dibromoanthranilic acid (**1**) [61] with cyclohexanone in POCl_3_ through an adapted reported procedure [62] (Scheme 1).

With precursor **2** in hand, we intended to expand the π-conjugation of the tetrahydroacridine core by adding diversely substituted aryl groups using the Suzuki–Miyaura cross-coupling reaction [63–64]. As a model reaction, we studied the coupling between 2,4-dibromo-9-chloro-5,6,7,8-tetrahydroacridine (**2**) and phenylboronic acid (**3a**). After a thorough optimization using Pd(PPh_3_)_4_ as catalyst, toluene as solvent, and K_3_PO_4_ as base, the desired bis-arylated product **4a** was isolated in up to 91% yield. The trifold Suzuki–Miyaura coupling product, i.e., 2,4,9-triphenyl-5,6,7,8-tetrahydroacridine, could not be prepared, even after increasing the amount of phenylboronic acid to 4 equivalents and prolonging the reaction time.

Our primary screening consisted in evaluating the effect of the catalyst amount. Several attempts were performed showing that a low concentration of the catalyst was required to avoid the formation of elevated amounts of homocoupling and side products. We found that reducing the amount of Pd(PPh_3_)_4_ from 5 mol % to 1 mol % gave higher yields of 2,4-diphenyl-9-chloro-5,6,7,8-tetrahydroacridine (**4a)** (Table 1, entries 1–6). Towards a better understanding of the regioselectivity of the coupling, we carried out a series of experiments with decreased amounts of phenylboronic acid. In all cases, the cross-coupling took place at both bromine atoms of **4a** and we could never isolate the corresponding monoarylated coupling product. The replacement of Pd(PPh_3_)_4_ by PdCl_2_(PPh_3_)_2_ afforded **4a** in almost the same yield (Table 1, entries 5 and 7). Concerning the impact of the base, we found that the application of K_2_CO_3_ or Cs_2_CO_3_ instead of K_3_PO_4_ did not lead to an improvement of the yield (Table 1, entries 8 and 9). However, increasing the amount of K_3_PO_4_ to 4 equivalents gave a nearly quantitative yield (Table 1, entry 12). We believe that a supplementary activation of the boronic acid, by adding a high amount of base, improved the nucleophilicity and facilitated the transmetalation. The use of dioxane instead of toluene (Table 1, entry 11) gave again a very good yield (89%). However, the employment of THF resulted in a decreased yield (Table 1, entry 10). The best result for the Suzuki–Miyaura cross-coupling between **2** and **3a** was obtained using 1 mol % of Pd(PPh_3_)_4_ and 4 equivalents of K_3_PO_4_ in toluene at 100 °C for 4 hours.

**Table 1:** Optimization of the Suzuki–Miyaura coupling between **2** and **3a**.^a^

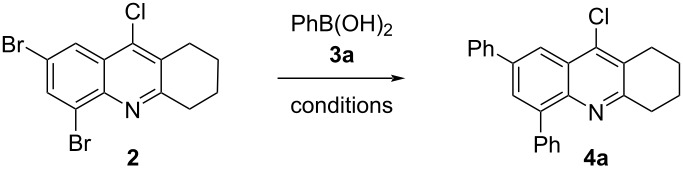					

entry	catalyst	[mol %]	base	solvent	yield^b^ (%)

1	Pd(PPh_3_)_4_	5	K_3_PO_4_	toluene	60
2	Pd(PPh_3_)_4_	4	K_3_PO_4_	toluene	67
3	Pd(PPh_3_)_4_	3	K_3_PO_4_	toluene	71
4	Pd(PPh_3_)_4_	2	K_3_PO_4_	toluene	82
5	Pd(PPh_3_)_4_	1	K_3_PO_4_	toluene	91
6	Pd(PPh_3_)_4_	0.5	K_3_PO_4_	toluene	84
7	PdCl_2_(PPh_3_)_2_	1	K_3_PO_4_	toluene	90
8	Pd(PPh_3_)_4_	1	K_2_CO_3_	toluene	88
9	Pd(PPh_3_)_4_	1	Cs_2_CO_3_	toluene	87
10	Pd(PPh_3_)_4_	1	K_3_PO_4_	THF	75
11	Pd(PPh_3_)_4_	1	K_3_PO_4_	dioxane	89
12	Pd(PPh_3_)_4_	1	K_3_PO_4_ (4 equiv)	toluene	95

^a^Reagents and conditions: catalyst, base, **2** (0.5 mmol), **3a** (1.1 mmol), solvent (5 mL), 100 °C, 4 h; ^b^isolated yield.

**Scheme 2:**
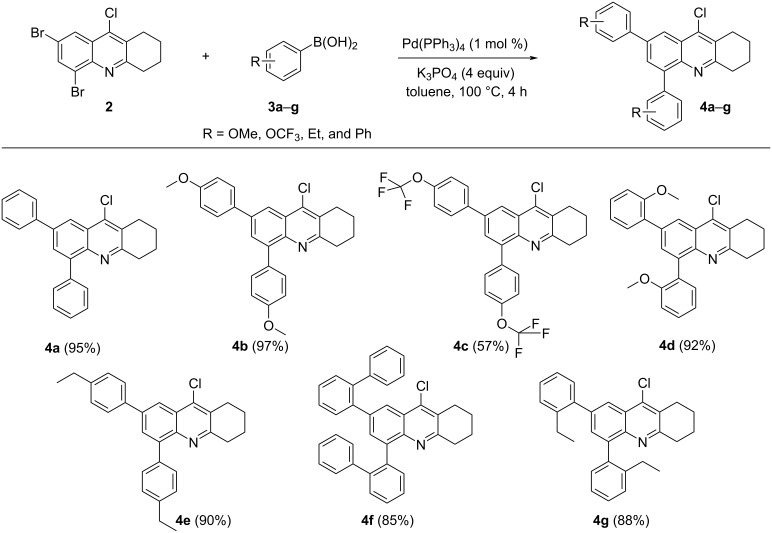
Synthesis of compounds **4a–g**.

With our optimized conditions in hand, we examined the scope of the reaction of **2** with other arylboronic acids **3b–g** (Scheme 2).

As expected, we found that electron-donating groups located in the arylboronic acid improved the yield. The cross-coupling of **2** with arylboronic acids **3b** and **3d,** bearing a methoxy group in position 2 or 4, afforded the corresponding products **4b** and **4d** in excellent yields. Arylboronic acids containing an alkyl group in different positions afforded good yields for products **4e** and **4g**. With the substrate containing a trifluoromethoxy group (OCF_3_), a longer reaction time of 6 hours was required to obtain the product **4c** in decreased yield (57%).

**Photophysical properties**. We studied the steady-state absorption and emission of selected tetrahydroacridines **4a–d** to gain insights into their photophysical properties. The UV–vis absorption spectra were measured in diluted dichloromethane solutions (*c* = 1 × 10^−5^ M) at room temperature. Table 2 summarizes the obtained spectral data, fluorescence quantum yields, stokes shifts, and optical band gap energies.

**Table 2:** Photophysical properties of tetrahydroacridines **4a–d** in dichloromethane solution.

	emission					absorption			
			
compound	λ_em_(nm)	FWHM^a^	ϕ_fluo_^b^ (%)	ν̃ stokes^c^		λ_abs_ (nm )	log ε	λ_onset_ (nm)	*E*_g_^opt^ (eV)^d^

**4a**	393	57	1.1	4854		329	4.88	367	3.37
**4b**	410	61	8.2	5730		332	5.07	387	3.20
**4c**	406	67	1.5	3778		352	511	401	3.09
**4d**	409	62	7.8	5580		333	5.05	389	3.18

^a^Spectrum full width at half maximum (nm); ^b^fluorescence standard: quinine bisulfate in 1 N H_2_SO_4_ (ϕ_fluo_ = 0.54); ^c^Stokes shift in wavenumber (cm^−1^) = (1/λ_abs_^max^ − 1/λ_em_^max^)·10^7^; ^d^estimated from the onset point of the absorption spectra: *E*_g_^opt^ = 1240/λ_onset_.

**Figure 2:**
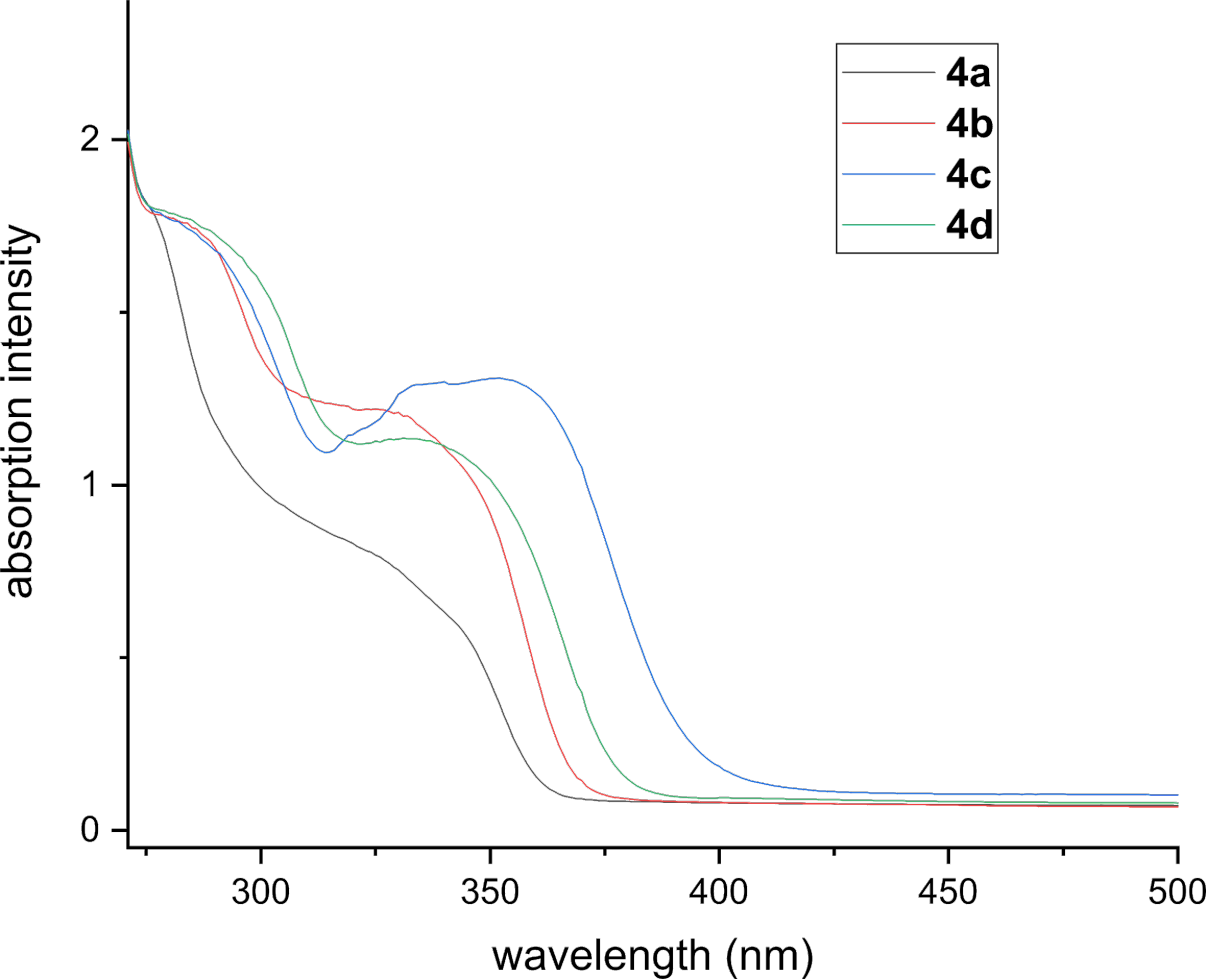
UV–vis absorption spectra of **4a–d** at room temperature in dilute dichloromethane solutions (*c* = 1 × 10^−5^ M).

As shown in Figure 2, most of the compounds show a broad band at around 320–360 nm in the UV region assigned to the π–π* electronic transitions. The phenyl unsubstituted derivative **4a** exhibited a main wide band with a shoulder peak at 329 nm. Derivatives **4b** and **4d**, bearing methoxy substituents, showed similar optical absorptions to those of **4a** with slight red shifts. In case of the trifluoromethoxy-substituted derivative **4c**, a larger red shift of 23 nm was observed. The optical gap energies of tetrahydroacridines **4a–d** were estimated from the onset point of the absorption spectra [65]. The parent derivative **4a** shows an onset of absorption at 363 nm and its optical band gap was deduced to be around 3.37 eV. Derivatives **4b** and **4d** showed similar values of 3.20 eV and 3.18 eV, respectively. In contrast, compound **4c** showed a decreased value of 3.09 eV.

**Figure 3:**
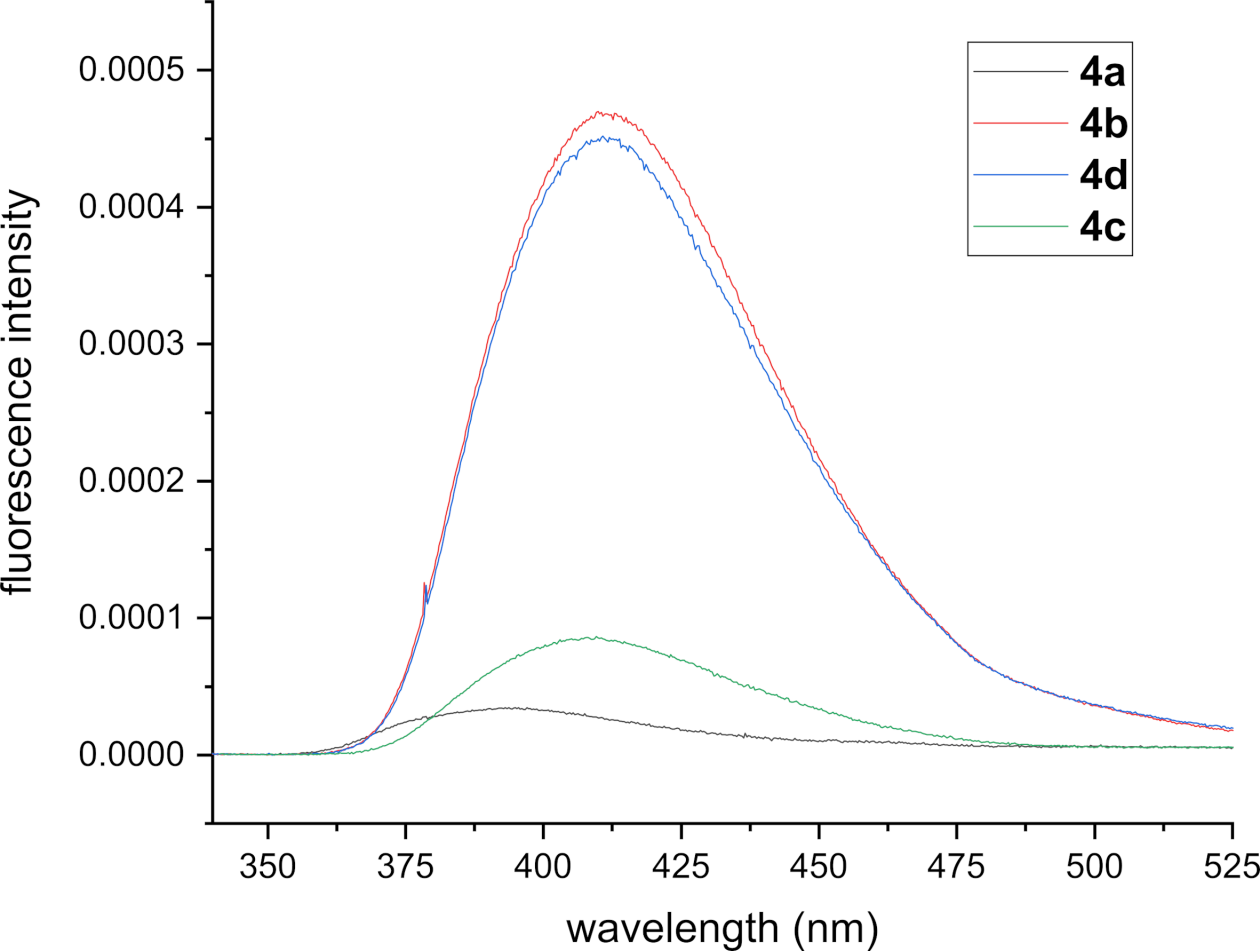
Fluorescence spectra of **4a–d** at room temperature in dilute dichloromethane solutions (*c* = 1 × 10^−5^ M).

The emission spectra of tetrahydroacridines **4a–d** were measured with an excitation line at 325 nm. They were dominated by broad bands ranging from 365 to 500 nm (Figure 3). The emission spectrum of **4a** appeared as a blue emission at 393 nm. Trifluoromethoxy derivative **4c** exhibited a red shift of 10 nm as compared to **4a**. Methoxy derivatives **4b** and **4d** showed similar emission bands at around 410 nm and gave larger red shifts. Their bands at lower energy may be attributed to the intramolecular charge transfer (ICT) from the electron-donating methoxy groups to the tetrahydroacridine core.

The Stokes shifts of **4a–d** were calculated based on their absorption and emission spectra. Derivative **4b**, containing an electron-donating methoxy substituent in *para*-position, gave the highest value of 5770 cm^−1^. The fluorescence quantum yields of **4a–d** were calculated with a comparative method, where quinine sulfate (SQ) in 0.1 M H_2_SO_4_ was used as standard [66].

**Figure 4:**
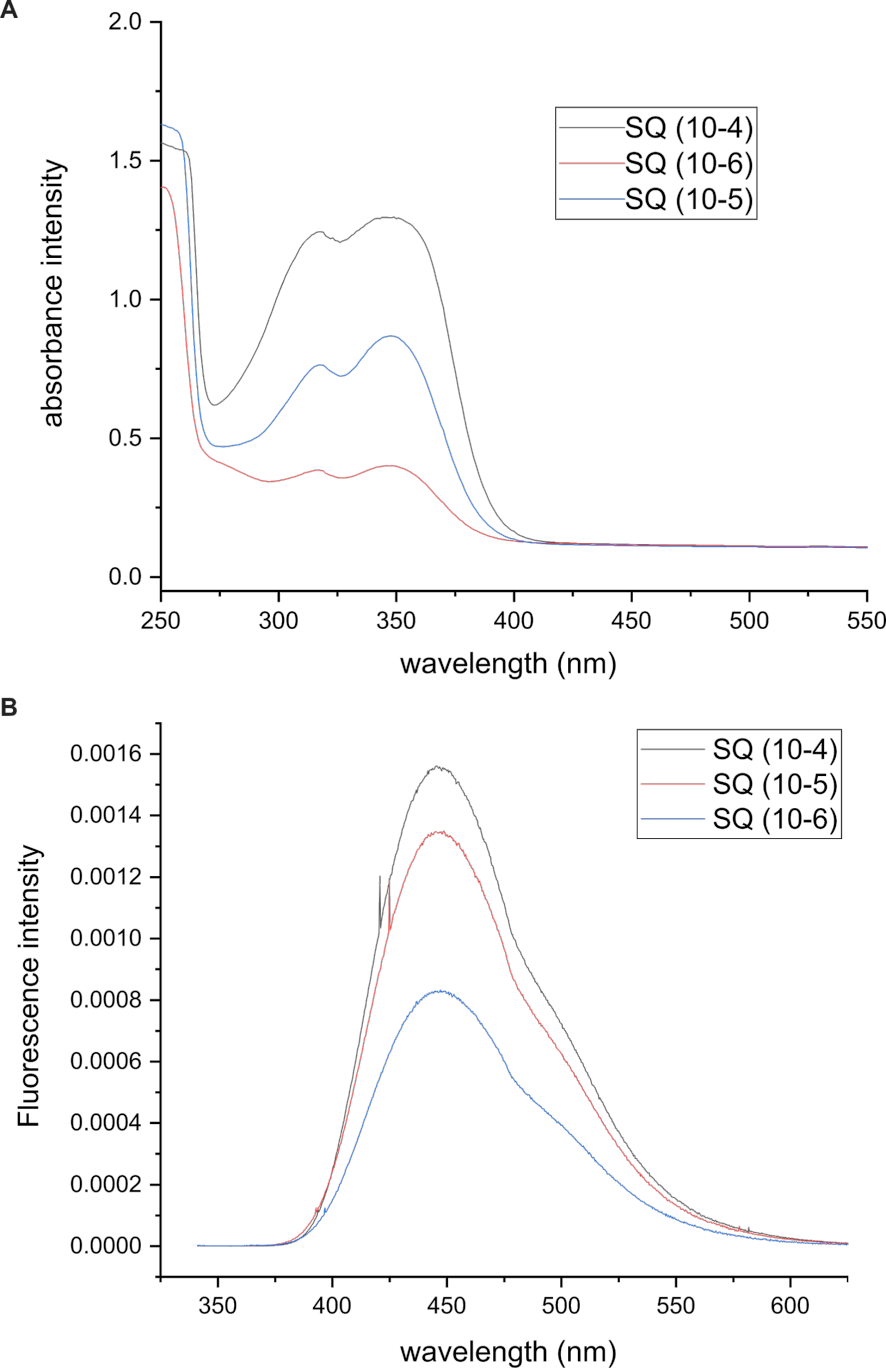
A) Absorbance and B) emission spectra for the standard quinine sulfate (SQ).

**Figure 5:**
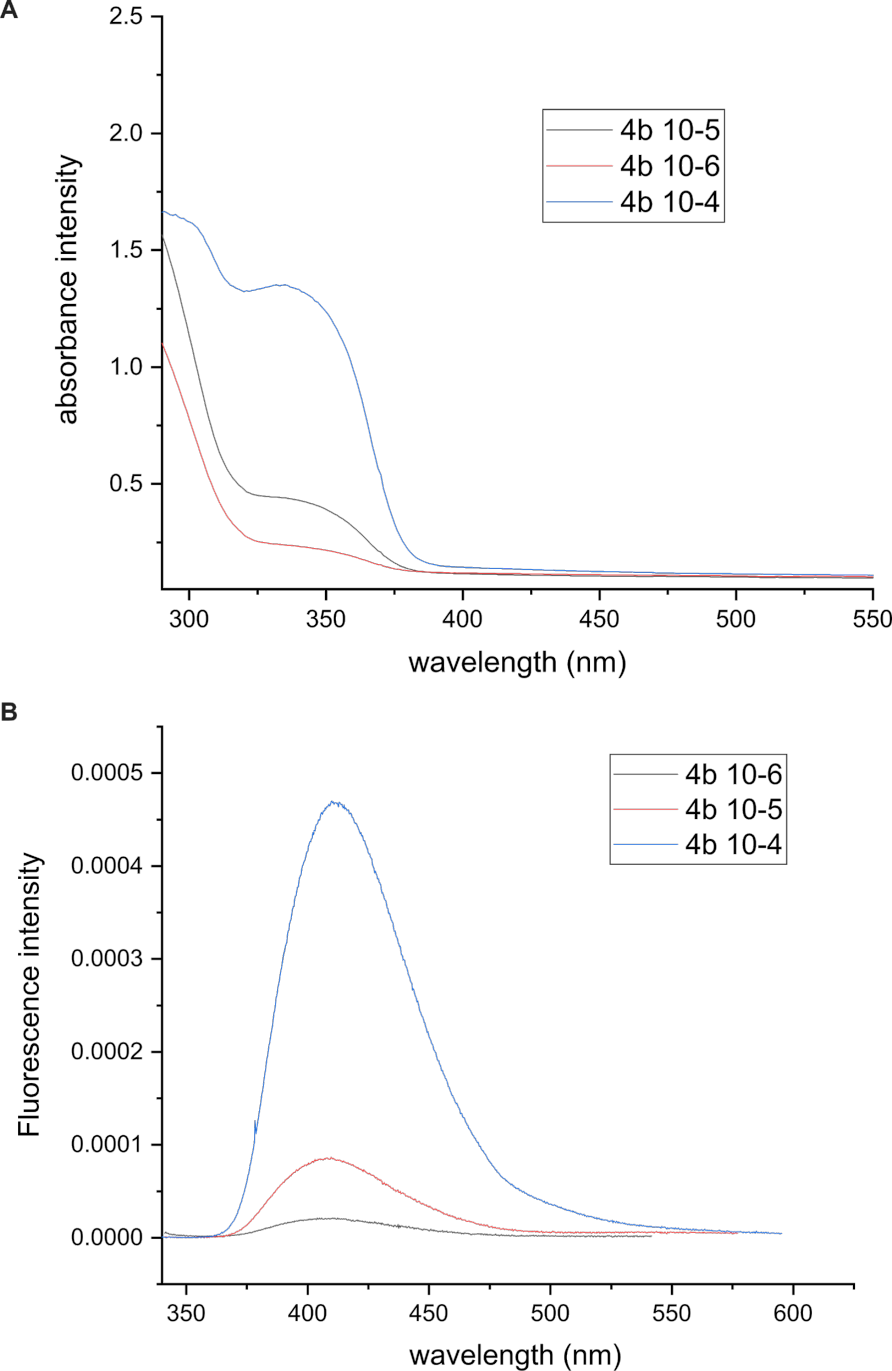
A) Absorbance and B) fluorescence spectra for **4b**.

**Figure 6:**
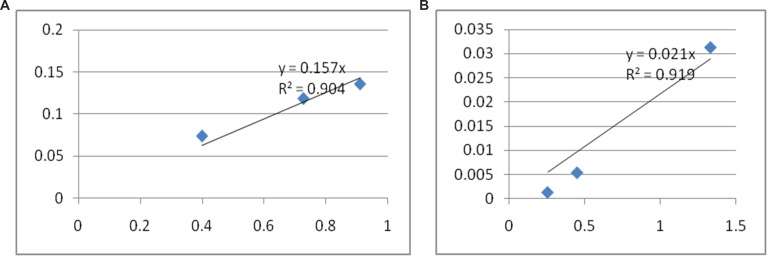
A) Plot of fluorescence intensities against their absorbances for quinine sulfate (SQ) and B) plot of fluorescence intensities against their absorbances for compound **4b**.

The fluorescence and absorbance spectra for quinine sulfate and product **4b** are given in Figure 4 and Figure 5, respectively. In addition, their plots of fluorescence intensities against absorbances are shown in Figure 6A and 6B. The tetrahydroacridine derivative **4b** gave the highest fluorescence quantum yield of 8.2%.

**Figure 7:**
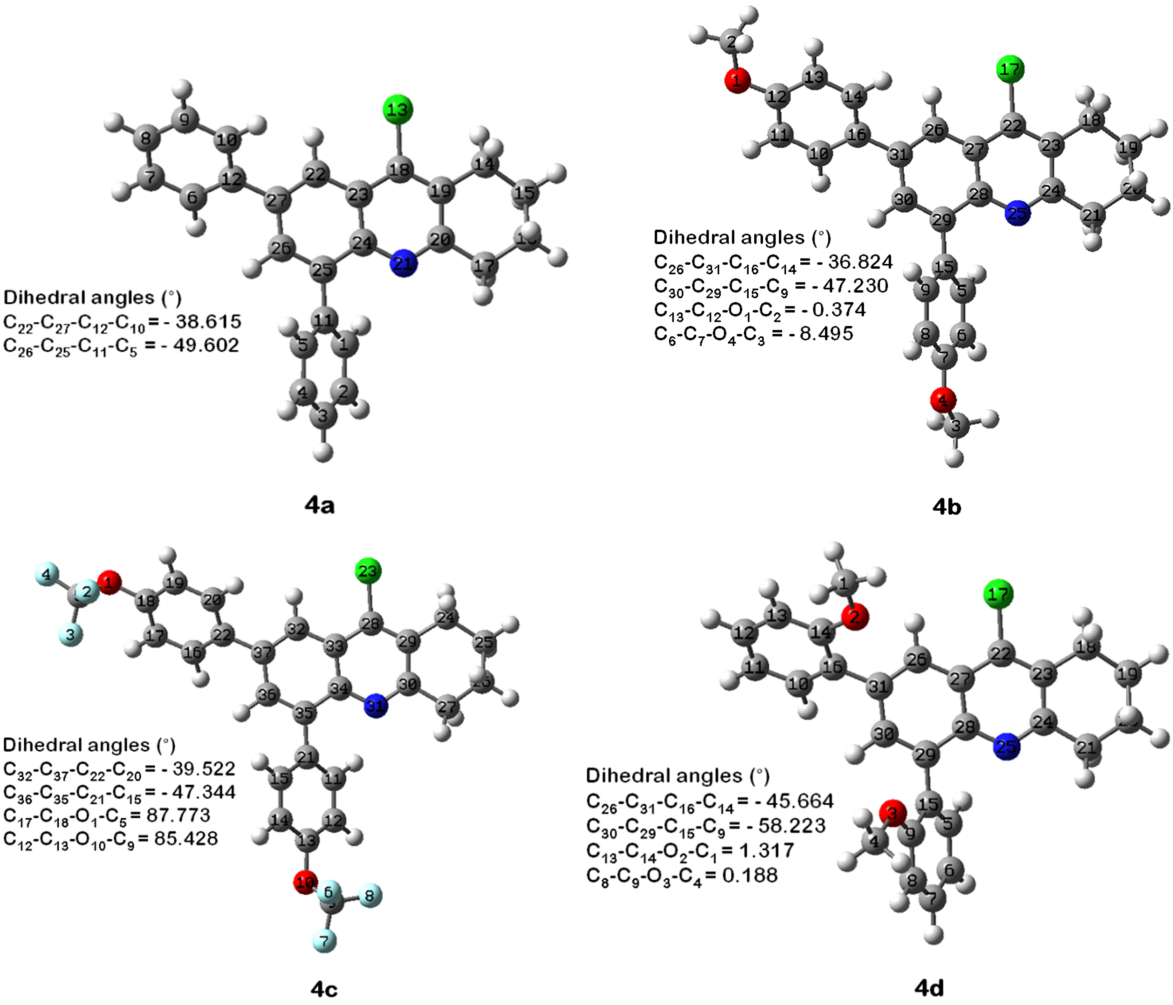
Selected dihedral angles (°) for compounds **4a–d**.

In order to elucidate the experimental observations and to further study the electronic properties, DFT calculations were carried out on the fully ground state at the restricted B3LYPlevel with 6-31G(d) basis set using dichloromethane as a continuum solvent model. The optimized chemical structures of **4a–d** are given in Figure 7 with selected geometrical parameters including dihedral angles and bonding and non-bonding distances (Table 3). It can be seen from Figure 7 that the four obtained structures are non-planar, due to steric hindrance and electronic repulsions. For compound **4a**, considerable distortions of −38.61 and −49.60 degrees between the tetrahydroacridine plane and the two phenyl groups were observed, due to rotations around the C-2 and C-4 single bonds. These torsion angles did not change significantly for products **4b** and **4c** bearing two substituents located at the *para*-positions. However, when the substituents were methoxy groups located at the *ortho*-positions, the dihedral angles were larger with values of −45.66 and −58.22 degrees, presumably due to steric reasons. These geometries may affect the electronic parameters of tetrahydroacridines **4a–d** and particularly their band gap energies.

**Table 3:** Selected bonding and non-bonding distances (Ǻ) for compounds **4a–d**.

	**4a**	**4b**	**4c**	**4d**

bonding distances (Å)	C_27_–C_12_ = 1.4853 C_25_–C_11_ = 1.4883	C_31_–C_16_ = 1.4833 C_29_–C_15_ = 1.4862	C_37_–C_22_ = 1.4850 C_35_–C_21_ = 1.4879	C_31_–C_16_ = 1.4884 C_29_–C_15_ = 1.4906
non-bonding distances (Å)	C_22_–C_10_ = 3.0201 C_26_–C_5_ = 3.0036 N–C_1_ = 2.9923	C_26_–C_14_ = 3.0200 C_30_–C_9_ = 2.9963 N–C_5_ = 2.9778	C_32_–C_20_ = 3.0229 C_36_–C_15_ = 3.0021 N–C_11_ = 2.9782	C_26_–C_14_ = 3.1294 C_30_–C_9_ = 3.1216 N–C_5_ = 3.0248

**Figure 8:**
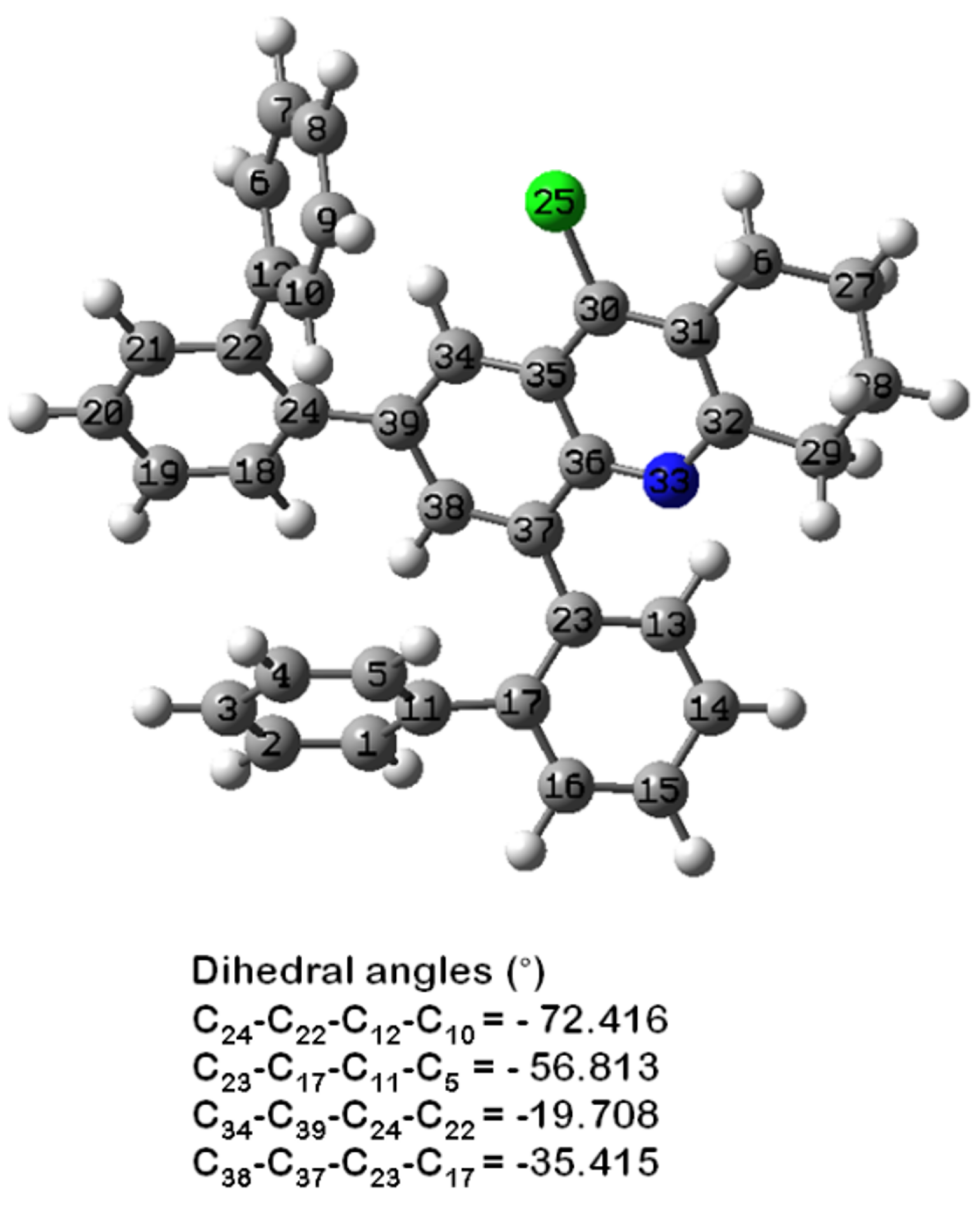
Selected dihedral angles (°) for compound **4f**.

On the other hand, the photophysical properties of products **4e–g** were not significantly affected by the aryl substituent. Electronic effects of the alkyl group were, as expected, very weak and no significant additional distortions arise from a *para*- or *ortho*-ethyl group for products **4e** and **4g**. Therefore, they gave the same absorption and emission as the parent product **4a**. As well, the phenyl *ortho*-substituent in the aryl ring for **4f** was twisted out of the plane and was not involved in the electronic orbital distribution (Figure 8).

**Figure 9:**
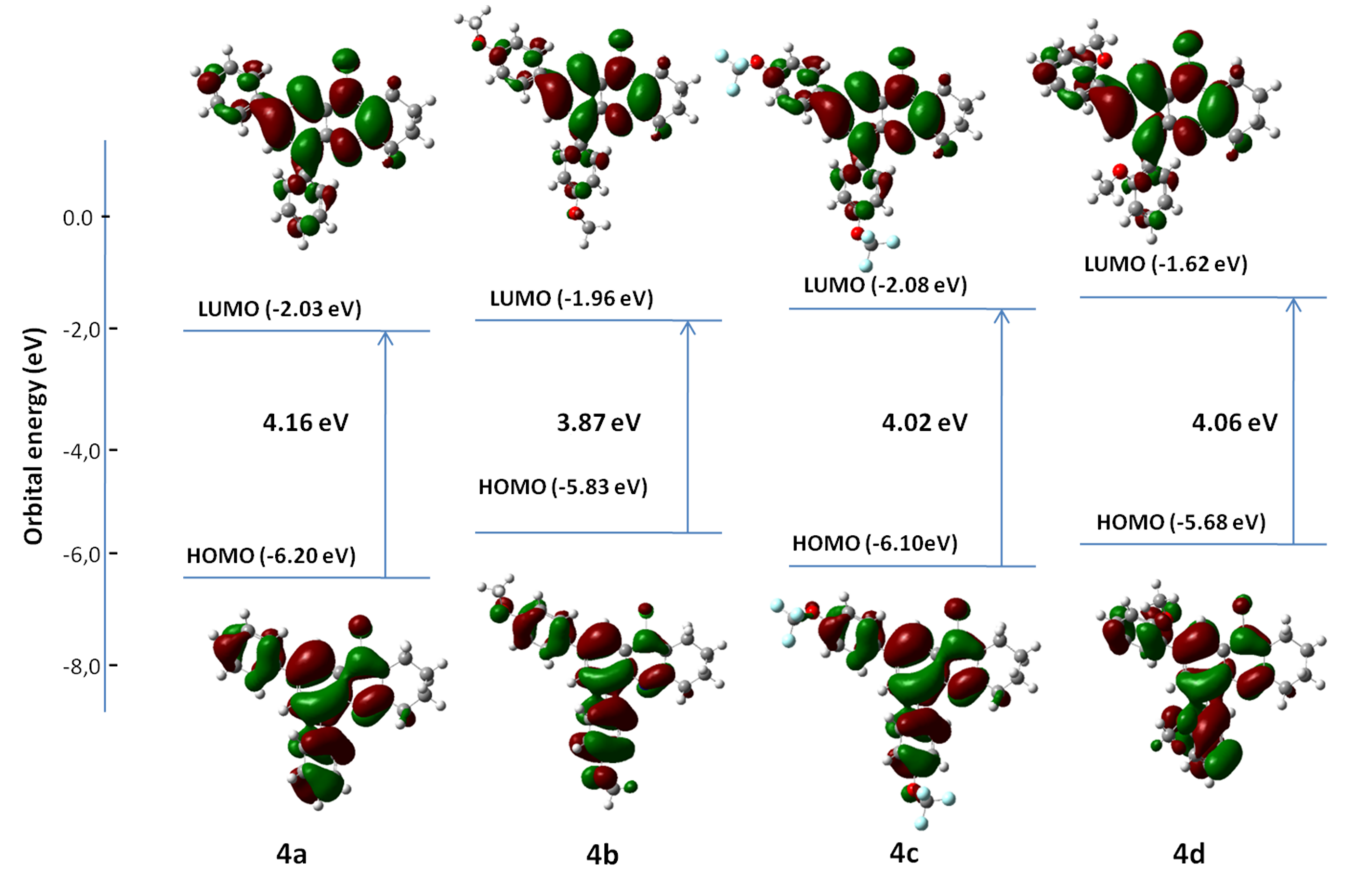
Calculated energy levels for compounds **4a–d** and their spatial distribution of the HOMO–LUMO frontier molecular orbitals from DFT calculations.

As shown in Figure 9, the HOMO and LUMO frontier orbitals of products **4a–d** are extended over the π-conjugated part of the molecules and, as expected, the cyclohexane ring is not involved. In all cases, the electron densities of HOMO were localized in the π-bonding orbitals between the carbon backbone of the quinoline ring and its two external phenyls. The LUMO electron densities were mainly located in the π* antibonding orbitals outside the carbon cores. It was observed for compound **4c** that the two trifluoromethoxy groups were twisted out of the plane and consequently did not participate in the orbital distribution. The HOMO electronic distributions of **4b** and **4d** were spread over the two non-twisted methoxy groups.

The calculated values of HOMO, LUMO, and the band gap energies of compounds **4a**–**d** at the B3LYP level of theory with 6-31G(d) basis set using dichloromethane as a continuum solvent model are given in Figure 9. The energy gap values are affected by the type and the position of the substituent located at the phenyl groups. In fact, different values of 3.87 eV and 4.06 eV were observed for compounds **4b** and **4d** having a methoxy group at the *ortho*- or *para*-positions, respectively. The electron-withdrawing trifluoromethoxy groups in compound **4c** lead to a decrease in the HOMO energy to −6.10 eV and LUMO energy to −1.86 eV giving an energy gap of 4.02 eV. The HOMO energy of derivative **4b** was the highest and as a result it exhibited the lowest value of *E*_gap_ and its electron conductivity was the best.

**Figure 10:**
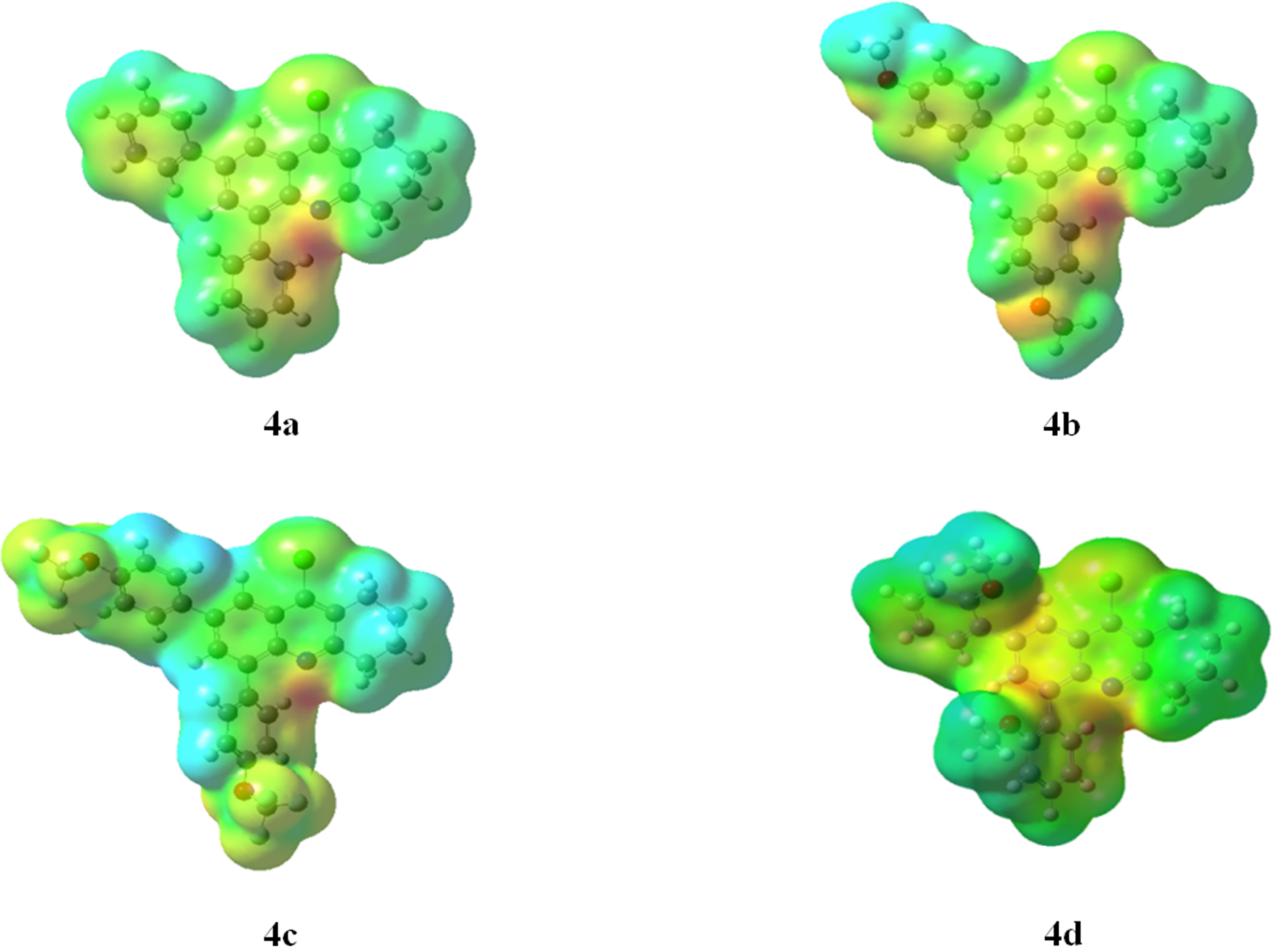
Visualization of MEP for compounds **4a–d** calculated by B3LYP method with 6-31G(d) basis set.

The molecular electrostatic potential maps for tetrahydroacridines **4a–d** are shown in Figure 10. The red and blue colors visualize the region of the attractive and repulsive potential, respectively. For the phenyl-substituted derivative **4a**, the blue-colored surface, located mainly at the cyclohexane ring, visualizes the electron deficiency (high electrostatic potential). The red regions, localized essentially at the nitrogen atom and at the two external phenyl groups, show the electron abundance (low electrostatic potential). The trifluoromethoxy group for **4c** resulted in a decrease of electron density in the tetrahydroacridine core. In addition, this strong electron-withdrawing effect induced the appearance of blue surfaces around the phenyl groups leading to a significant decrease in their electronic densities. However, thanks to the π-donating effect of two methoxy groups for **4b** and **4d**, yellow-red regions are present in the phenyl groups and quinoline core.

**Figure 11:**
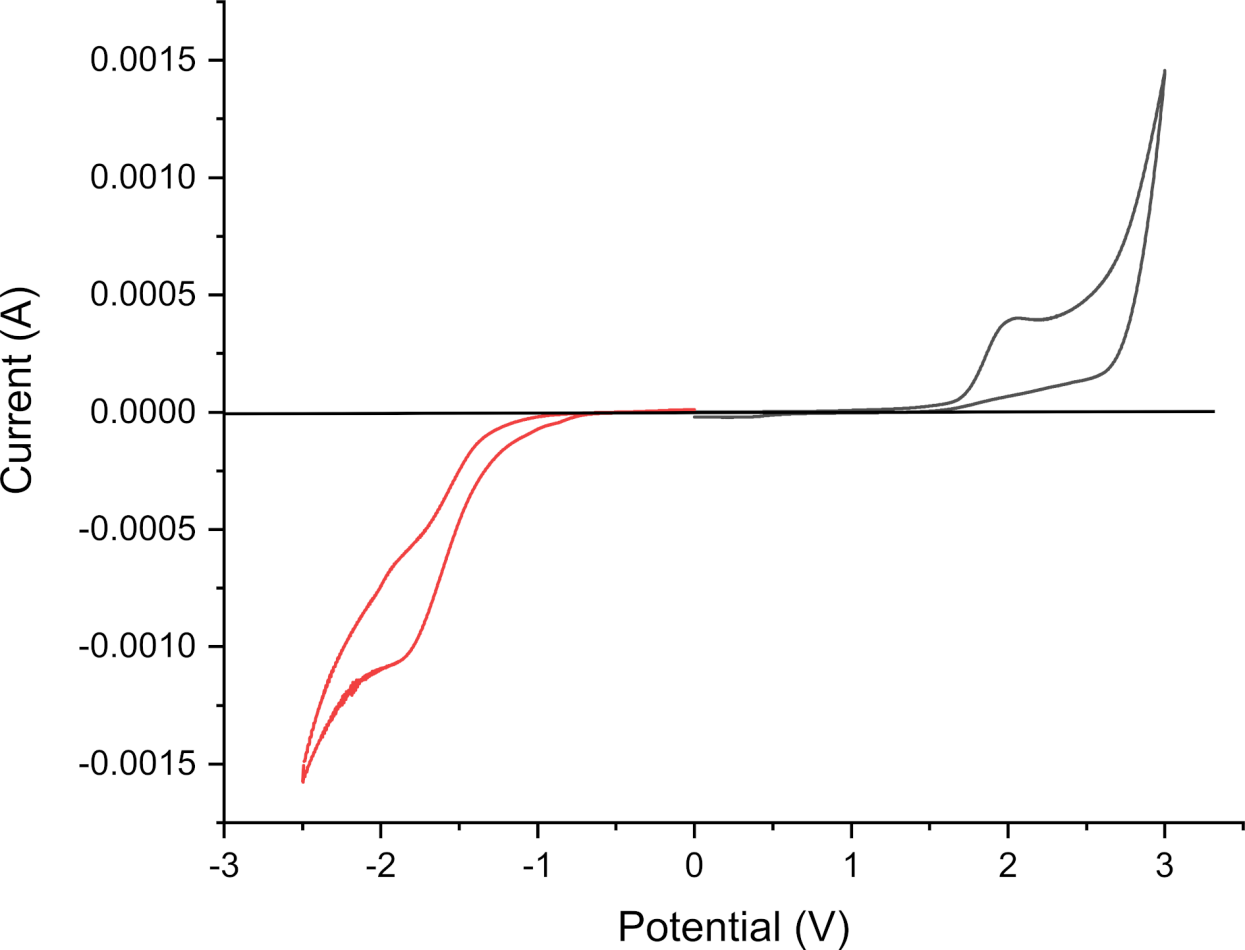
Cyclic voltammogram for **4c** in 0.1 M (*n*-Bu)_4_NBF_4_/acetonitrile at a scan rate of 50 mV/s.


The electrochemical behavior of compound **4c** was studied by cyclic voltammetry (CV) in dry acetonitrile. The obtained voltammogram, recorded at a scanning rate of 50 mV/s, is given in Figure 11. The HOMO and LUMO energy levels were deduced from the onset potentials of the oxidation and reduction waves, respectively. The potential of the saturated Ag/AgCl reference electrode was calibrated using the ferrocene/ferrocenium (Fc/Fc+) redox system [67], the HOMO and LUMO energies were calculated by using the following equations:

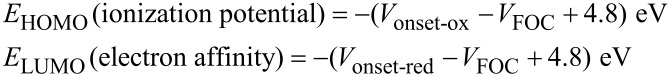


Here, *V*_FOC_ is the potential value of Fc/Fc+ under the same experimental conditions (0.52 V) and 4.8 eV is the HOMO energy value of Fc/Fc+ with respect to zero vacuum level. The onset potentials for the oxidation and reduction of **3c** were 1.50 eV and −1.36 eV, respectively. Thus, their LUMO and the HOMO energies were found to be −2.92 eV and −5.78 eV, respectively, giving rise to an electrochemical band gap of 2.86 eV. We noticed a difference of 0.23 eV between the optical and the electrochemical band gap which is attributed to the interface barrier between the electrode and the tetrahydroacridine, as well as to the exciton binding energy [68–69]. On the other hand, the calculated HOMO energy was more negative than the experimental value, while the calculated LUMO energy was less negative. This can be explained by the fact that the calculations were achieved based on an equilibrium conformer.

## Conclusion

In conclusion, we developed a convenient synthesis of novel 2,4-diaryl-9-chloro-5,6,7,8-tetrahydroacridines starting from commercially available anthranilic acid. Their photophysical properties show an impact of the aryl substituents on absorption and fluorescence. Methoxy substituents lead to a red shift of the emission and exhibited the highest fluorescence quantum yields. According to the theoretical studies, all products adopted a distorted molecular geometry. In contrast to methoxy groups, the trifluoromethoxy groups are twisted out of plane and, therefore, are not involved in the electronic orbital distribution. Experimental energy band gaps of the prepared tetrahydroacridines were found to be an average of 3 eV which makes them promising candidates for optoelectronic applications.

## Experimental

### Materials and measurements

Anhydrous solvents and chemicals were purchased from Sigma–Aldrich and used without further purification. All reactions were carried out under an inert argon atmosphere and were monitored by thin-layer chromatography (TLC). Organic compounds were purified using commercial Merck silica gel (0.043–0.06 mm) with a fluorescence indicator (the visualization was performed under UV light at 254 nm). All solvents for work-up and column chromatography were distilled before use. NMR data were recorded in CDCl_3_ (tetramethylsilane as the internal standard) on Bruker ARX 300 instruments. Signals due to the solvent are: CHCl_3_: δ 7.26 for ^1^H and δ 77.16 for ^13^C. Peak characterization of ^1^H NMR spectra: s = singlet, d = doublet, t = triplet, q = quartet, m = multiplet. Chemical shifts were given in ppm (δ) relative to tetramethylsilane (SiMe_4_) as internal standard. High-resolution MS (HRMS–ESI) was performed on an Agilent 1969 A TOF. The photophysical studies were carried out in freshly prepared dichloromethane solutions with concentrations of 1 × 10^−5^ M. The UV–vis spectra were recorded on a Shimadzu 2401 PC spectrophotometer in quartz cuvettes with a path length of 1 cm. Emission spectra were recorded on a Perkin-Elmer LS 50B spectrofluorimeter. Cyclic voltammetry was performed in anhydrous acetonitrile solution containing 0.1 M of tetrabutylammonium tetrafluoroborate (*n-*Bu_4_NBF_4_) as a supporting electrolyte by using a three-electrode cell with a glassy carbon electrode as the working electrode, a platinum wire as the counter electrode, and an Ag/AgCl electrode as the reference electrode. All the measurements were scanned in negative and positive directions at a scan rate of 50 mV/s, as controlled by a PGSTAT30 Autolab potentiostat.

### Theoretical calculations

The theoretical studies were realized in vacuum with the Gaussian 09 program [70]. The geometry of the equilibrium conformer at the ground state was first found at AM1 level. Then, further optimization through density functional theory (DFT) approach [71] at the restricted Becke, 3-parameter, Lee–Yang–Parr hybrid functional (B3LYP) with standard basis set 6-31G (d) were carried out.

### Experimental procedure for the Suzuki–Miyaura coupling and spectroscopic data for 9-chloro-2,4-diaryl-5,6,7,8-tetrahydroacridine derivatives **4a–g**

2,4-Dibromo-9-chloro-5,6,7,8-tetrahydroacridine (**2**, 372.89 mg, 1 mmol, 1 equiv), phenylboronic acid (268.24 mg, 2.2 mmol, 2.2 equiv), Pd(PPh_3_)_4_ (11.55 mg, 0.010 mmol, 1 mol %), and K_3_PO_4_ (849 mg, 4 mmol, 4 equiv) were added to a dried glass pressure tube. The tube was evacuated and backfilled three times with argon, then toluene (5 mL) was added. The tube was sealed with a Teflon cap and heated to 100 °C for 4 h until completion of the reaction (reaction progress was monitored by TLC). The mixture was then cooled to room temperature and the solvent was removed under reduced pressure. Water (10 mL) was added and extracted using CH_2_Cl_2_ (3 × 10 mL). The combined organic layers were dried (Na_2_SO_4_) and the solvent was evaporated under reduced pressure. The crude product was purified by column chromatography using heptanes/ethyl acetate 9:1.

**2,4-Diphenyl-9-chloro-5,6,7,8-tetrahydroacridine (4a):** white solid; 95%; mp 139–141 °C; ^1^H NMR (300 MHz, CDCl_3_) δ 1.71–1.89 (m, 4H, 2CH_2_), 2.84–3.05 (m, 4H, 2CH_2_), 7.25–7.50 (m, 6H, aryl-H), 7.66–7.71 (m, 4H, aryl-H), 7.91 (s, 1H, aryl-H), 8.31 (s, 1H, aryl-H); ^13^C NMR (75 MHz, CDCl_3_) δ 22.6 (CH_2_), 22.7 (CH_2_), 27.6 (CH_2_), 34.3 (CH_2_), 121.0 (C_Ar_), 126.0 (C_Ar_), 127.4 (C_Ar_), 127.5 (C_Ar_), 127.8 (C_Ar_), 127.8 (C_Ar_), 128.9 (C_Ar_), 129.0 (C_Ar_), 130.0 (C_Ar_), 131.0 (C_Ar_), 138.8 (Cl-C_Ar_), 139.2 (C_Ar_), 140.3 (C_Ar_), 140.4 (C_Ar_), 141.8 (C_Ar_), 143.4 (N-C_Ar_), 159.1 (N =C_Ar_); HRMS–ESI (*m*/*z*): [M + H]^+^ calcd for C_25_H_20_ClN, 370.1355; found, 370.1357.

**2,4-Bis(4-methoxyphenyl)-9-chloro-5,6,7,8-tetrahydroacridine (4b):** yellowish solid; (Hep/EA 9:1), 97%; mp 135–137 °C; ^1^H NMR (300 MHz, CDCl_3_) δ 1.91–1.99 (m, 4H, 2CH_2_), 3.08–3.12 (m, 4H, 2CH_2_), 3.88 (s, 3H, OCH_3_), 3.92 (s, 3H, OCH_3_), 7.01–7.10 (m, 4H, aryl-H), 7.76–7.88 (m, 4H, aryl-H), 7.92 (s, 1H, aryl-H), 8.33 (s, 1H, aryl-H); ^13^C NMR (75 MHz, CDCl_3_) δ 22.7 (CH_2_), 22.8 (CH_2_), 26.5 (CH_2_), 34.4 (CH_2_), 55.3 (OCH_3_), 55.4 (OCH_3_), 113.3 (C_Ar_), 114.4 (C_Ar_), 119.7 (C_Ar_), 126.1 (C_Ar_), 128.5 (C_Ar_), 128.8 (C_Ar_), 129.3 (C_Ar_), 131.8 (C_Ar_), 132.2 (C_Ar_), 133.0 (C_Ar_), 138.4 (Cl-C_Ar_), 139.8 (C_Ar_), 141.4 (C_Ar_), 143.5 (C_Ar_), 158.5 (O-C_Ar_), 159.1 ( O-C_Ar_), 159.5 (N=C_Ar_); HRMS–ESI (*m*/*z*): [M + H]^+^ calcd for C_27_H_24_ClNO_2_, 430.1496; found, 430.1498.

**2,4-Bis(4-trifluoromethoxyphenyl)-9-chloro-5,6,7,8-tetrahydroacridine (4c):** white solid; (Hep/EA 9:1), 57%; mp 127–129 °C; ^1^H NMR (300 MHz, CDCl_3_) δ 1.71–1.89 (m, 4H, 2CH_2_), 2.91–2.99 (m, 4H, 2CH_2_), 7.11–7.27 (m, 4H, aryl-H), 7.66–7.88 (m, 4H, aryl-H), 7.72 (s, 1H, aryl-H), 8.23 (s, 1H, aryl-H); ^13^C NMR (75 MHz, CDCl_3_) δ 22.5 (CH_2_), 22.6 (CH_2_), 27.6 (CH_2_), 34.5 (CH_2_), 120.2 (C_Ar_), 121.5 (C_Ar_), 122.27 (CF_3_O), 122.34 (CF_3_O), 126.0 (C_Ar_), 128.4 (C_Ar_), 128.8 (C_Ar_), 129.7 (C_Ar_), 132.0 (C_Ar_), 136.5 (C_Ar_), 137.3 (C_Ar_), 138.5 (C_Ar_), 139.1 (Cl-C_Ar_), 141.5 (C_Ar_), 142.8 (C_Ar_), 143.0 (C_Ar_), 148.7 (O-C_Ar_), 149.8 (O-C_Ar_), 159.7 (N=C_Ar_); HRMS–ESI (*m*/*z*): [M + H]^+^ calcd for C_27_H_18_ClF_6_NO_2_, 538.0930; found, 538.0932.

**2,4-Bis(2-methoxyphenyl)-9-chloro-5,6,7,8-tetrahydroacridine (4d):** yellowish paste; (Hep/EA 9:1), 92%; mp 77–79 °C; ^1^H NMR (300 MHz, CDCl_3_) δ 1.71–1.79 (m, 4H, 2CH_2_), 2.91–2.98 (m, 4H, 2CH_2_), 3.85 (s, 3H, OCH_3_), 3.88 (s, 3H, OCH_3_), 6.91–6.96 (m, 4H, aryl-H), 7.71–7.78 (m, 4H, aryl-H), 7.82 (s, 1H, aryl-H), 8.23 (s, 1H, aryl-H); ^13^C NMR (75 MHz, CDCl_3_) δ 22.7 (CH_2_), 22.7 (CH_2_), 27.6 (CH_2_), 34.4 (CH_2_), 55.3 (OCH_3_), 55.4 (OCH_3_), 113.7 (C_Ar_), 114.4 (C_Ar_), 119.7 (C_Ar_), 126.1 (C_Ar_), 128.5 (C_Ar_), 128.8 (C_Ar_), 129.3 (C_Ar_), 131.8 (C_Ar_), 132.1 (C_Ar_), 133.0 (C_Ar_), 138.4 (Cl-C_Ar_), 139.8 (C_Ar_), 141.5 (C_Ar_), 143.4 (C_Ar_), 158.5 (O-C_Ar_), 159.1 (O-C_Ar_), 159.5 (N=C_Ar_); HRMS–ESI (*m*/*z*): [M + H]^+^ calcd for C_27_H_24_ClNO_2_, 430.1495; found, 430.1493.

**2,4-Bis(4-ethylphenyl)-9-chloro-5,6,7,8-tetrahydroacridine (4e):** pale green solid; 90%; mp 120–122 °C; ^1^H NMR (300 MHz, CDCl_3_) δ 1.31–1.45 (m, 6H, 2CH_3_), 1.81–2.07 (m, 4H, 2CH_2_), 2.67–2.85 (m, 4H, 2CH_2_), 3.07–3.25 (m, 4H, 2CH_2_), 7.35–7.45 (m, 4H, aryl-H), 7.63–7.75 (m, 4H, aryl-H), 8.10 (s, 1H, aryl-H), 8.44 (s, 1H, aryl-H); ^13^C NMR (75 MHz, CDCl_3_) δ 15.6 (CH_3_), 15.7 (CH_3_), 22.7 (CH_2_), 22.8 (CH_2_), 27.6 (CH_2_), 28.6 (CH_2_), 28.8 (CH_2_), 34.5 (CH_2_), 120.5 (C_Ar_), 126.1 (C_Ar_), 127.0 (C_Ar_), 127.4 (C_Ar_), 127.5 (C_Ar_), 128.3 (C_Ar_), 128.8 (C_Ar_), 129.7 (C_Ar_), 131.1 (C_Ar_), 136.8 (C_Ar_), 138.0 (Cl-C_Ar_), 138.8 (C_Ar_), 140.4 (C_Ar_), 141.5 (C_Ar_), 143.1 (C_Ar_),144.0 (N-C_Ar_), 158.8 (N=C_Ar_); HRMS–ESI (*m*/*z*): [M + H]^+^ calcd for C_29_H_28_ClN, 426.1910; found, 426.1913.

**2,4-Bis(2-phenylphenyl)-9-chloro-5,6,7,8-tetrahydroacridine (4f):** yellowish solid; 85%; mp 94–96 °C; ^1^H NMR (300 MHz, CDCl_3_) δ 1.44–1.69 (m, 4H, 2CH_2_), 2.54–2.65 (m, 4H, 2CH_2_), 6.77–6.89 (m, 6H, aryl-H), 6.96–7.18 (m, 6H, aryl-H), 7.29–7.44 (m, 6H, aryl-H), 7.81 (s, 1H, aryl-H), 8.11 (s, 1H, aryl-H); ^13^C NMR (75 MHz, CDCl_3_) δ 22.5 (CH_2_), 22.6 (CH_2_), 27.6 (CH_2_), 35.5 (CH_2_), 119.8 (C_Ar_), 123.5 (C_Ar_), 123.9 (C_Ar_), 124.5 (C_Ar_), 125.0 (C_Ar_), 125.4 (C_Ar_), 125.8 (C_Ar_), 126.1 (C_Ar_), 126.9 (C_Ar_), 127.2 (C_Ar_), 127.6 (C_Ar_), 127.9 (C_Ar_), 128.1 (C_Ar_), 129.1 (C_Ar_), 129.9 (C_Ar_), 130.1 (C_Ar_), 130.7 (C_Ar_), 131.6 (C_Ar_), 134.2 (C_Ar_), 137.1 (C_Ar_), 138.5 (Cl-C_Ar_), 139.5 (C_Ar_), 140.1 (C_Ar_), 140.8 (C_Ar_), 141.5 (C_Ar_), 143.1 (N-C_Ar_), 158.2 (C_Ar_), 159.0 (C_Ar_), 160.6 (N=C_Ar_); HRMS–ESI (*m*/*z*): [M + H]^+^ calcd for C_37_H_28_ClN, 522.1910; found, 522.1912.

**2,4-Bis(2-ethylphenyl)-9-chloro-5,6,7,8-tetrahydroacridine (4g):** brown solid; 88%; mp 186–188 °C; ^1^H NMR (300 MHz, CDCl_3_) δ 1.35–1.47 (m, 6H, 2CH_3_), 1.79–1.95 (m, 4H, 2CH_2_), 2.62–2.83 (m, 4H, 2CH_2_), 3.11–3.29 (m, 4H, 2CH_2_), 7.25–7.35 (m, 4H, aryl-H), 7.61–7.71 (m, 4H, aryl-H), 8.12 (s, 1H, aryl-H), 8.43 (s, 1H, aryl-H); ^13^C NMR (75 MHz, CDCl_3_) δ 15.5 (CH_3_), 15.6 (CH_3_), 22.7 (CH_2_), 22.8 (CH_2_), 27.5 (CH_2_), 28.5 (CH_2_), 28.7 (CH_2_), 34.4 (CH_2_), 120.3 (C_Ar_), 126.0 (C_Ar_), 126.3 (C_Ar_),127.2 (C_Ar_), 127.1 (C_Ar_), 127.6 (C_Ar_), 128.2 (C_Ar_), 128.7 (C_Ar_), 129.0 (C_Ar_), 129.6 (C_Ar_), 131.1 (C_Ar_), 131.8 (C_Ar_), 136.8 (C_Ar_), 138.1 (Cl-C_Ar_), 138.2 (C_Ar_), 138.9 (C_Ar_), 140.5 (C_Ar_), 141.4 (C_Ar_), 143.4 (C_Ar_), 144.1 (N-C_Ar_), 158.9 (N=C_Ar_); HRMS–ESI (*m*/*z*): [M + H]^+^ calcd for C_29_H_28_ClN, 426.1910; found, 426.1914.

## Supporting Information

File 1 Synthesis and analytical data of starting compound **2** and copies of spectra for the synthesized compounds.
