# Supplementary material for: Synthesis and investigation on optical and electrochemical properties of 2,4-diaryl-9-chloro-5,6,7,8-tetrahydroacridines
Source: Beilstein J Org Chem. 2021 Sep 20;17:2450–61. doi: 10.3762/bjoc.17.162 (PMC8474071; doi:10.3762/bjoc.17.162)
Supplement: File 1 — Synthesis and analytical data of starting compound 2 and copies of spectra for the synthesized compounds. [file Beilstein_J_Org_Chem-17-2450-s001.pdf]

BEILSTEIN JOURNAL OF ORGANIC CHEMISTRY

Supporting Information

for

Synthesis and investigation on optical and electrochemical properties of 2,4-diaryl-9-chloro-5,6,7,8-tetrahydroacridines

Najeh Tka, Mohamed Adnene Hadj Ayed, Mourad Ben Braiek, Mahjoub Jabli and Peter Langer

Beilstein J. Org. Chem. **2021,** *17,* 2450–2461. doi:10.3762/bjoc.17.162

Synthesis and analytical data of starting compound 2 and copies of spectra for the synthesized compounds

License and Terms: This is a supporting information file under the terms of the Creative Commons Attribution License (https://creativecommons.org/licenses/by/4.0). Please note that the reuse, redistribution and reproduction in particular requires that the author(s) and source are credited and that individual graphics may be subject to special legal provisions.
The license is subject to the *Beilstein Journal of Organic Chemistry* terms and conditions: (https://www.beilstein-journals.org/bjoc/terms)

1. **Procedure for preparing the starting material 2,4-dibromo-9-chloro-5,6,7,8-tetrahydroacridine (2)**

3,5-Dibromoanthranilic acid (2.92 g,10 mmol, 1 equiv) and cyclohexanone (1.07 mL, 11 mmol, 1.1 equiv) were stirred in an ice bath. Then, 15 mL of $POCl_3$ were carefully added and the mixture was heated under reflux for 4 h. The mixture was cooled to room temperature and concentrated to give a slurry. The residue was diluted with dichloromethane, neutralized with aqueous $NaHCO_3$, and washed with brine. The organic layer was dried over anhydrous K_2CO_3 and concentrated to afford a yellow solid. It was recrystallized from acetone to give **2** as pale yellow solid (3.24 g, 87%).

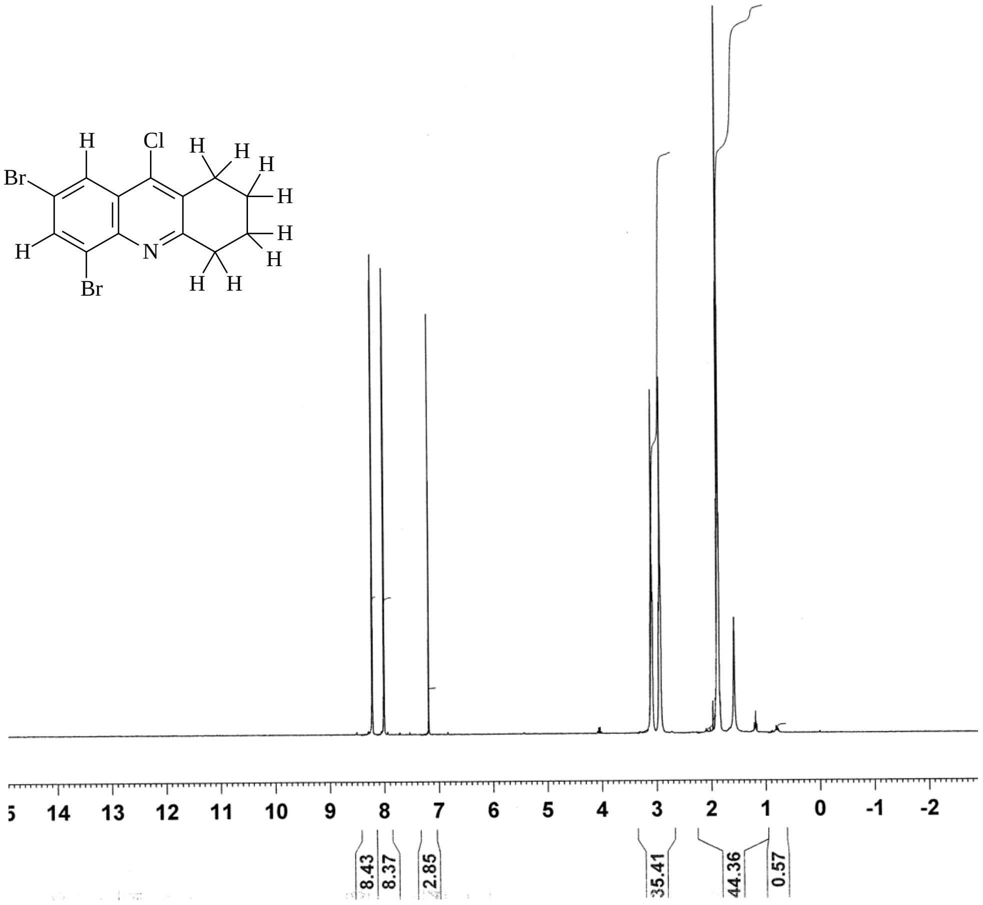

Figure S1: ^{1}H NMR (300 MHz, $CDCl_3$) of 2,4-dibromo-9-chloro-5,6,7,8-tetrahydroacridine (**2**).

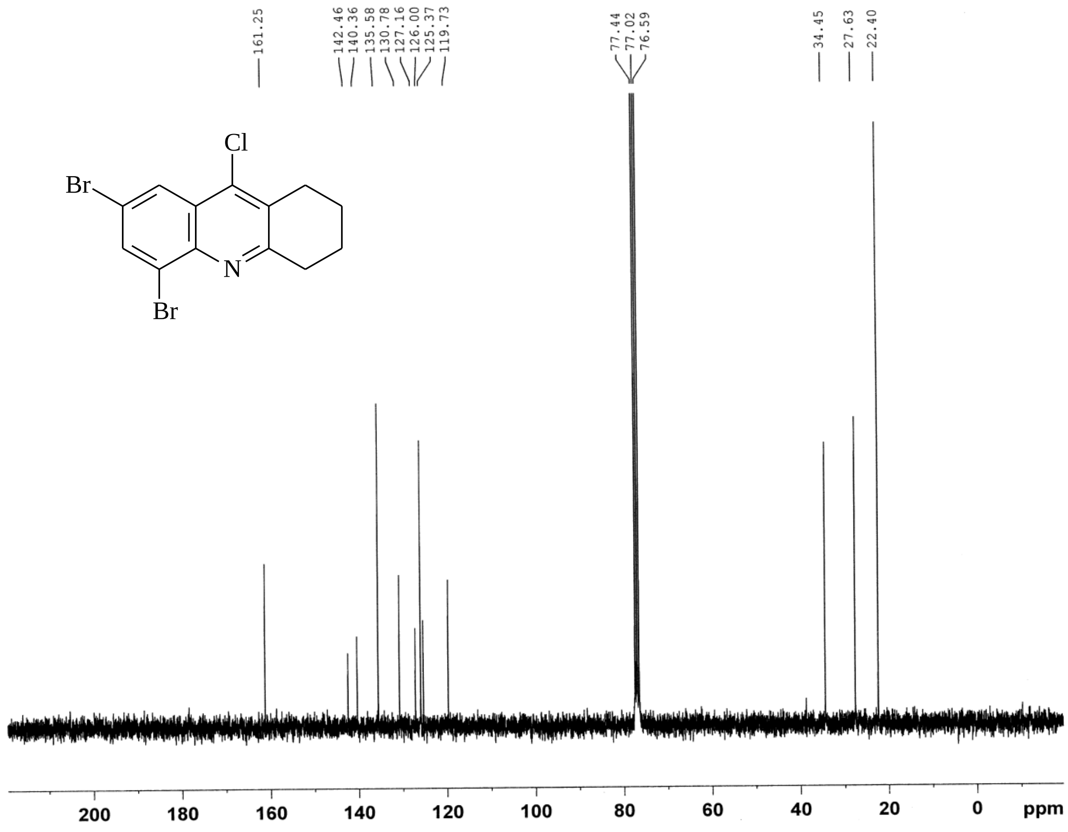

Figure S2: ^{13}C NMR (75 MHz, $CDCl_3$) of 2,4-dibromo-9-chloro-5,6,7,8-tetrahydroacridine (**2**).

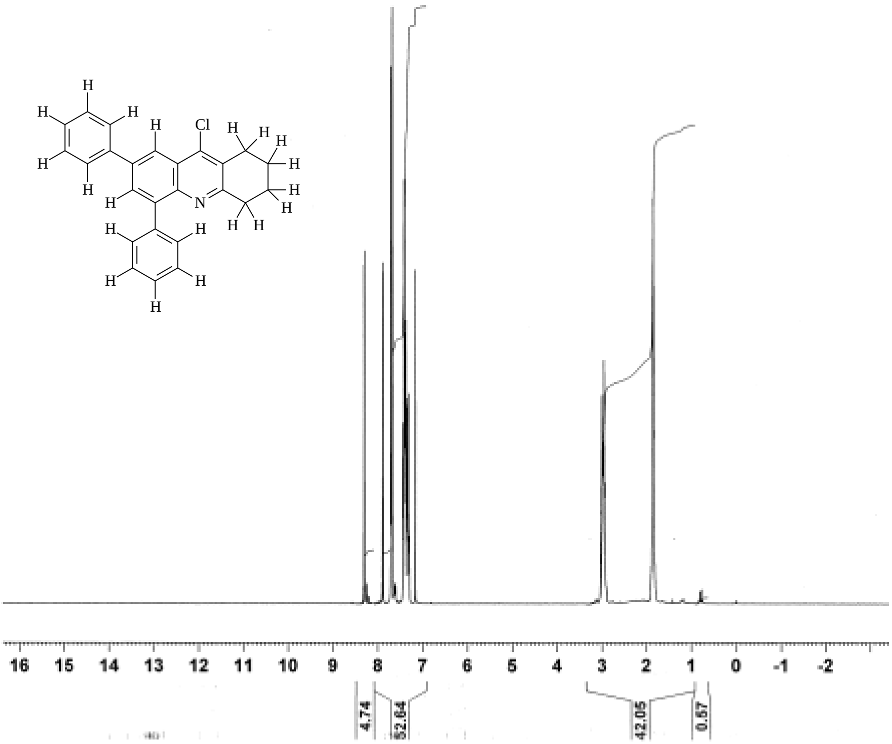

Figure S3: ^{1}H NMR (300 MHz, $CDCl_3$) of 2,4-diphenyl-9-chloro-5,6,7,8-tetrahydroacridine **(4a)**

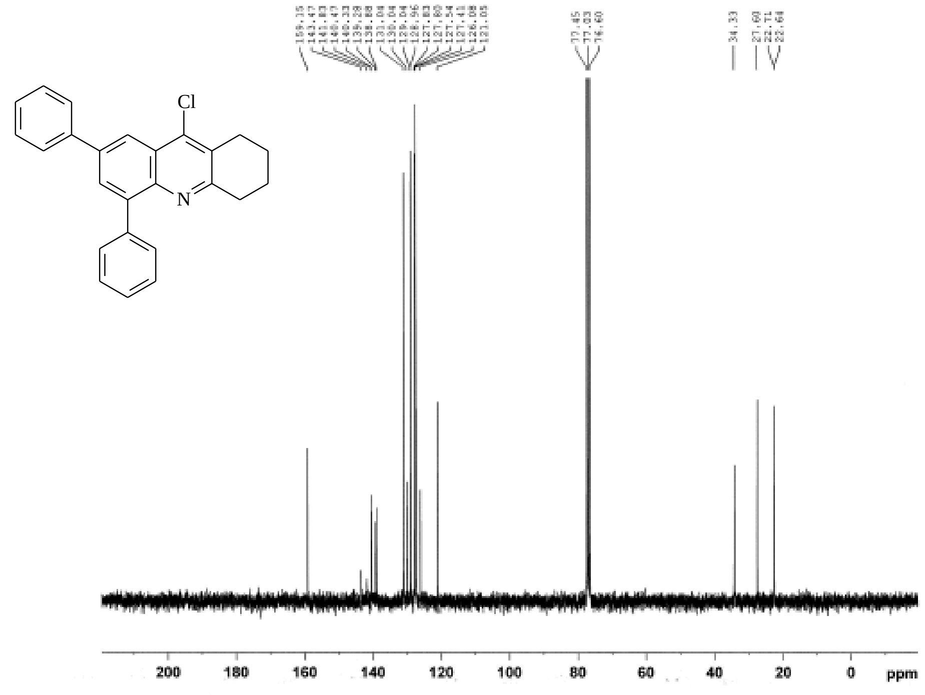

Figure S4: ^{13}C NMR (75 MHz, $CDCl_3$) of 2,4-diphenyl-9-chloro-5,6,7,8-tetrahydroacridine **(4a)**

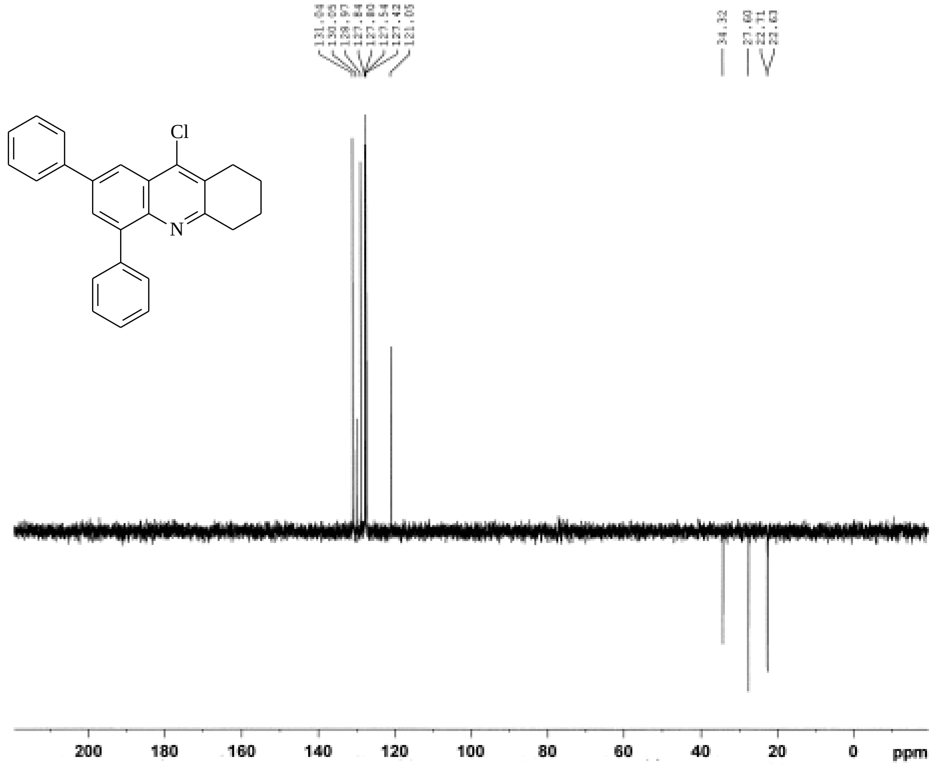

Figure S5: ^{13}C NMR DEPT 135 spectrum of 2,4-diphenyl-9-chloro-5,6,7,8-tetrahydroacridine **(4a)**

MS Zoomed Spectrum

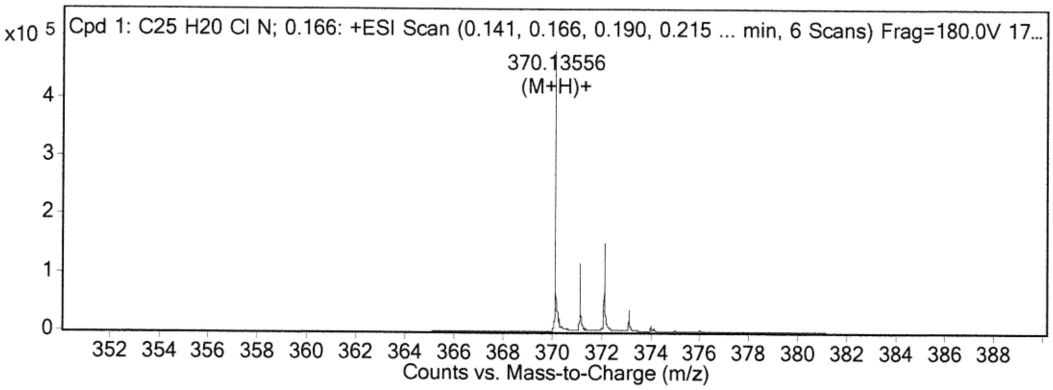

MS Spectrum Peak List

Ion	Abund	Formula	Calculated Mass	Measured Mass	Difference	Diff (ppm)
(M+H)+	485856.59	C25H20ClN	370.1357	370.13556	0.14	0.38

--- End Of Report ---

Figure S6: MS spectra of of 2,4-diphenyl-9-chloro-5,6,7,8-tetrahydroacridine **(4a)**

calculated mass: 369.1357

measured mass: 369.1355

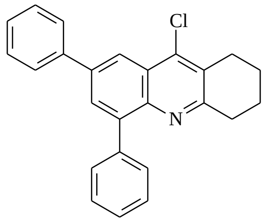

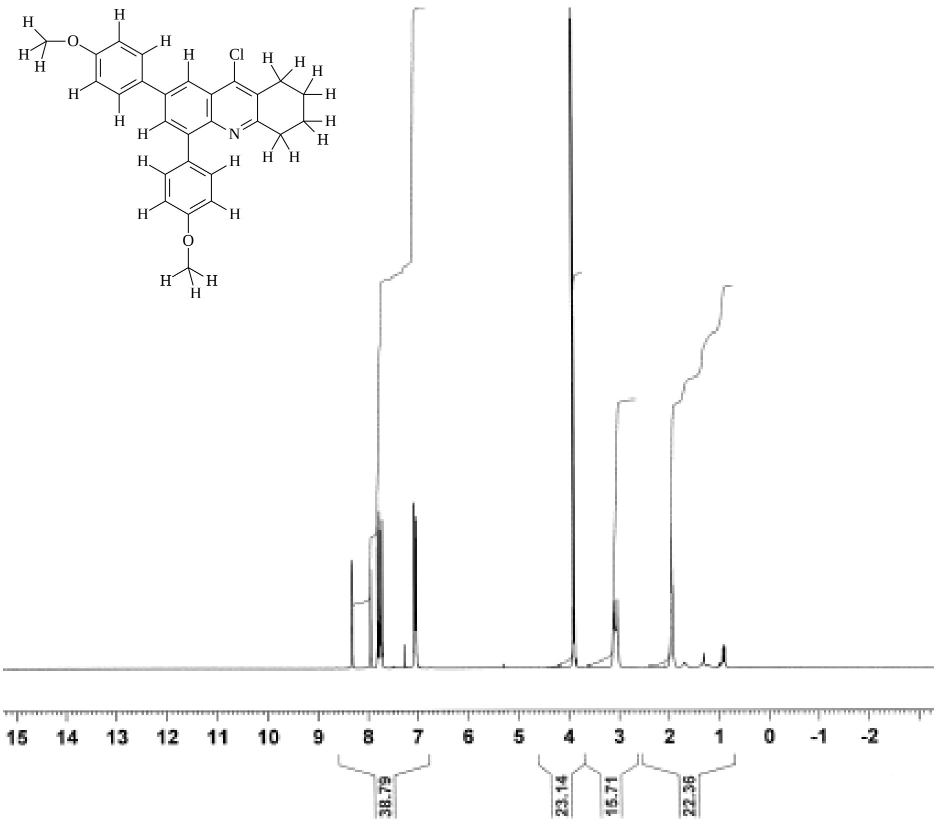

Figure S7: ^{1}H NMR (300 MHz, $CDCl_3$) of 2,4-bis(4-methoxyphenyl)-9-chloro-5,6,7,8-tetrahydroacridine **(4b)**

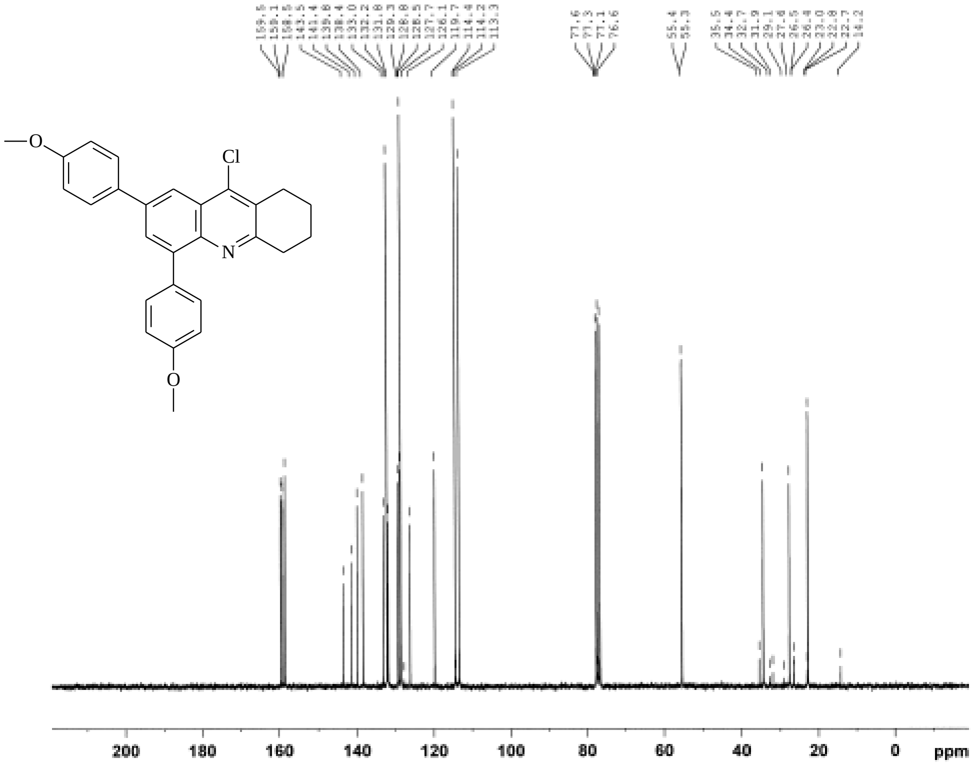

Figure S8: ^{13}C NMR (75 MHz, $CDCl_3$)) of 2,4-bis(4-methoxyphenyl)-9-chloro-5,6,7,8-tetrahydroacridine **(4b)**

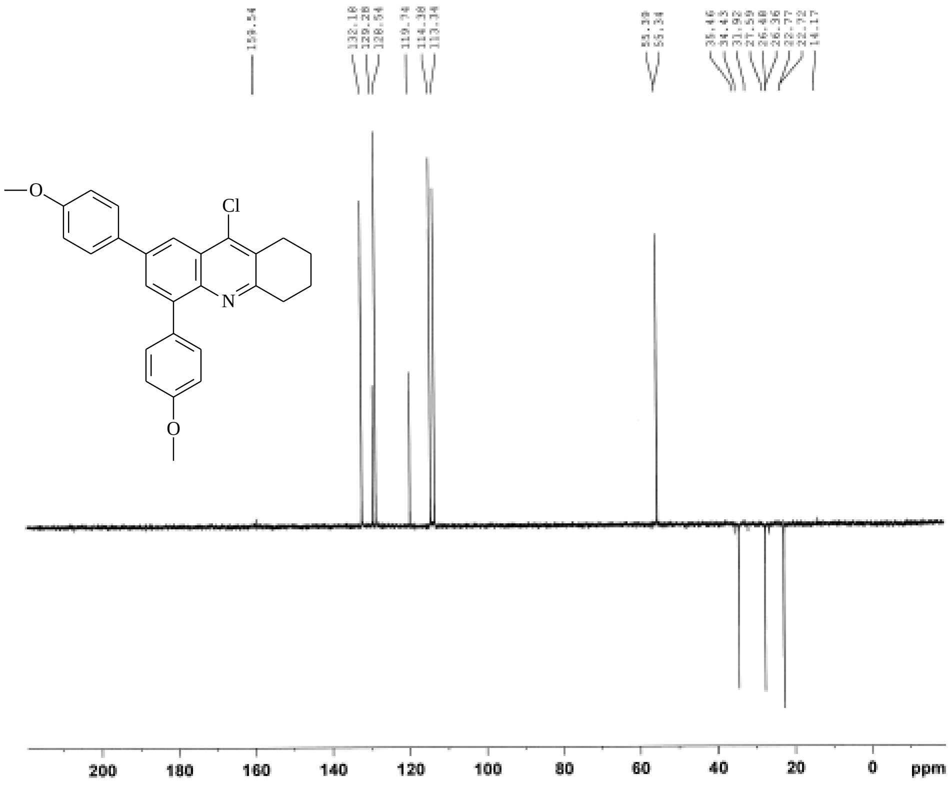

Figure S9: ^{13}C NMR DEPT 135 spectrum of 2,4-bis(4-methoxyphenyl)-9-chloro-5,6,7,8-tetrahydroacridine **(4b)**

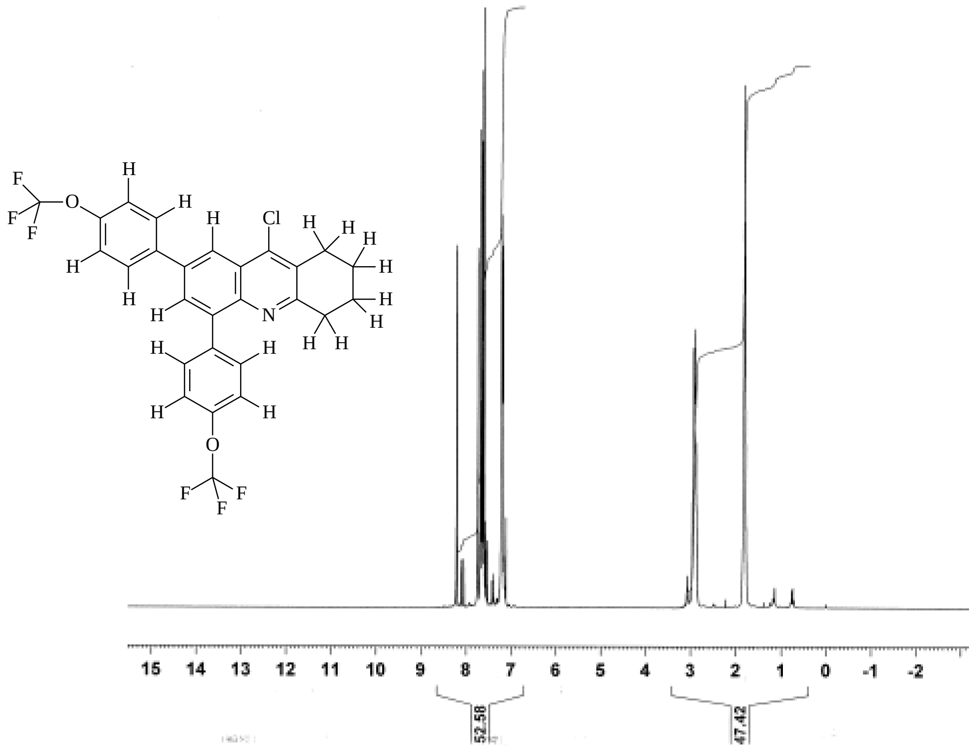

Figure S10: ^{1}H NMR (300 MHz, $CDCl_3$) of 2,4-bis(4-trifluoromethoxyphenyl)-9-chloro-5,6,7,8-tetrahydroacridine **(4c)**

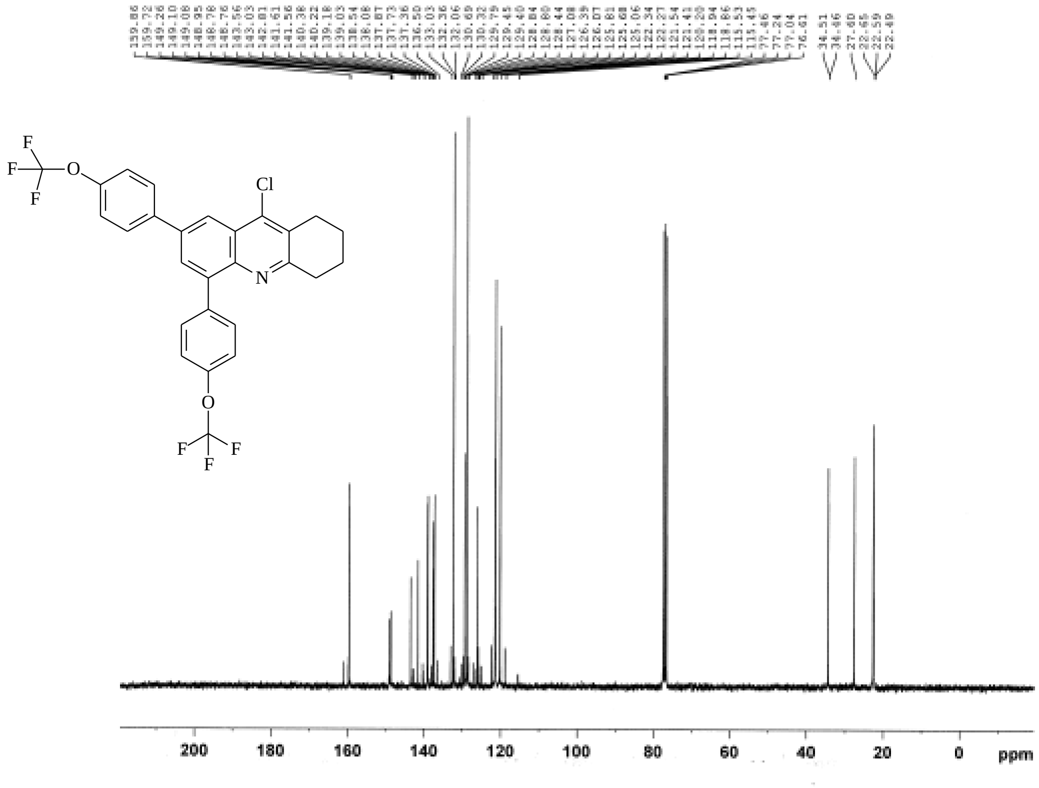

Figure S11: ^{13}C NMR (75 MHz, $CDCl_3$) of 2,4-bis(4-trifluoromethoxyphenyl)-9-chloro-5,6,7,8-tetrahydroacridine **(4c)**

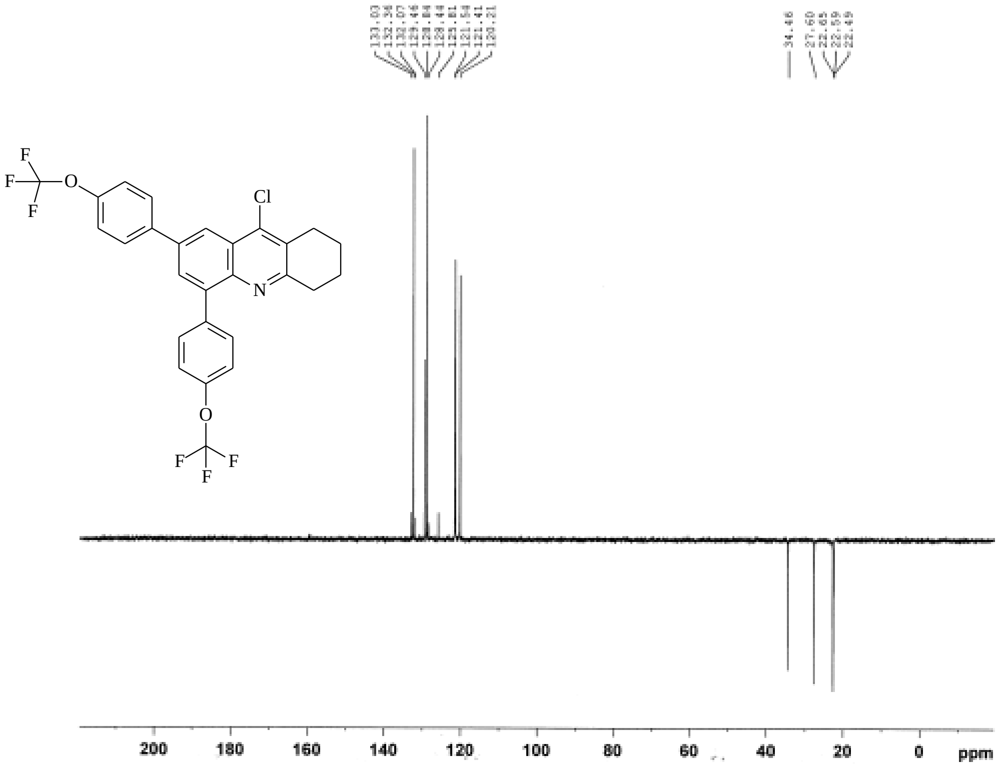

Figure S12: ^{13}C NMR DEPT 135 spectrum of 2,4-bis(4-trifluoromethoxyphenyl)-9-chloro-5,6,7,8-tetrahydroacridine **(4c)**

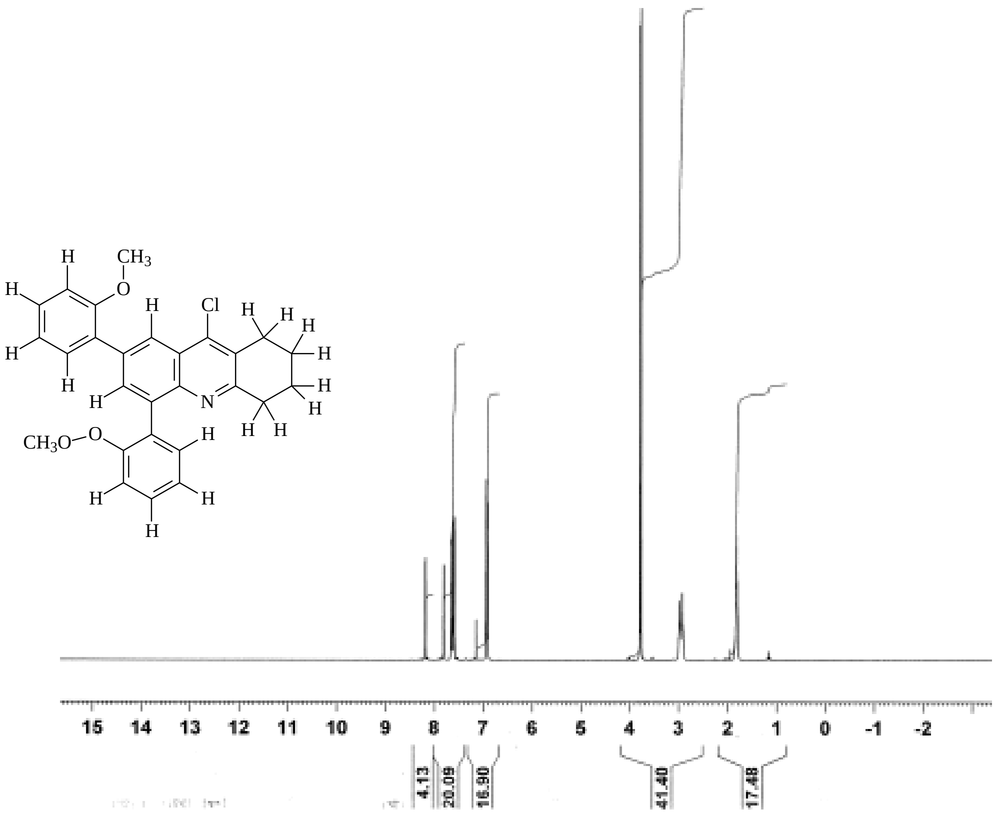

Figure S13: ^{1}H NMR (300 MHz, $CDCl_3$) of 2,4-bis(2-methoxyphenyl)-9-chloro-5,6,7,8-tetrahydroacridine **(4d)**

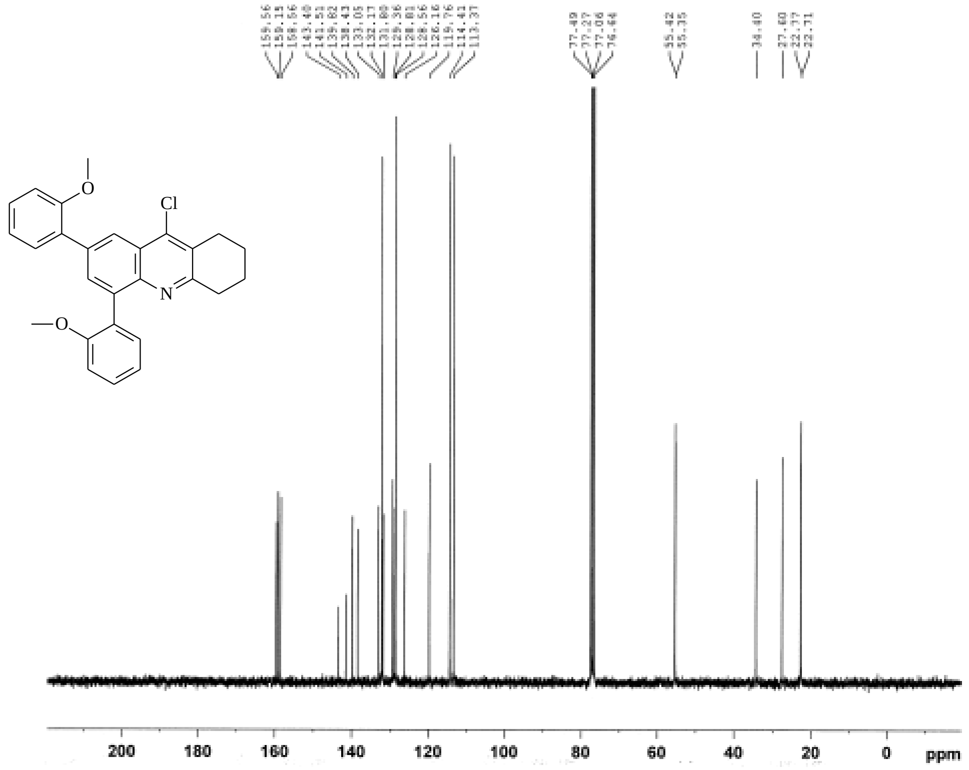

Figure S14: ^{13}C NMR (75 MHz, $CDCl_3$) of 2,4-bis(2-methoxyphenyl)-9-chloro-5,6,7,8-tetrahydroacridine **(4d)**

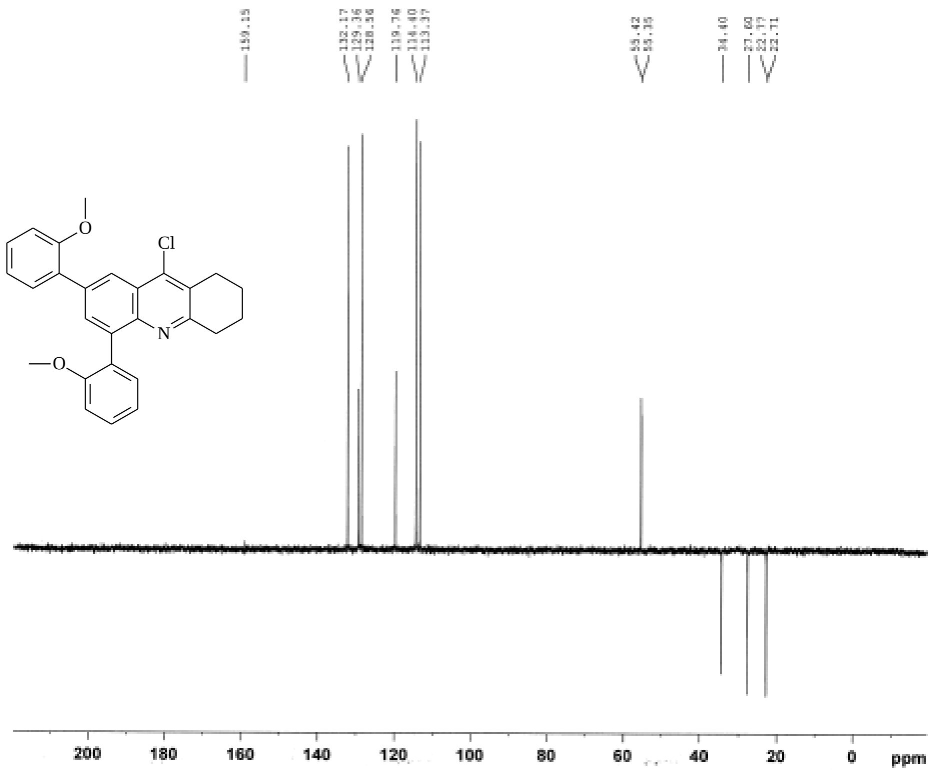

Figure S15: ^{13}C NMR DEPT 135 of 2,4-bis(2-methoxyphenyl)-9-chloro-5,6,7,8-tetrahydroacridine

(4d)

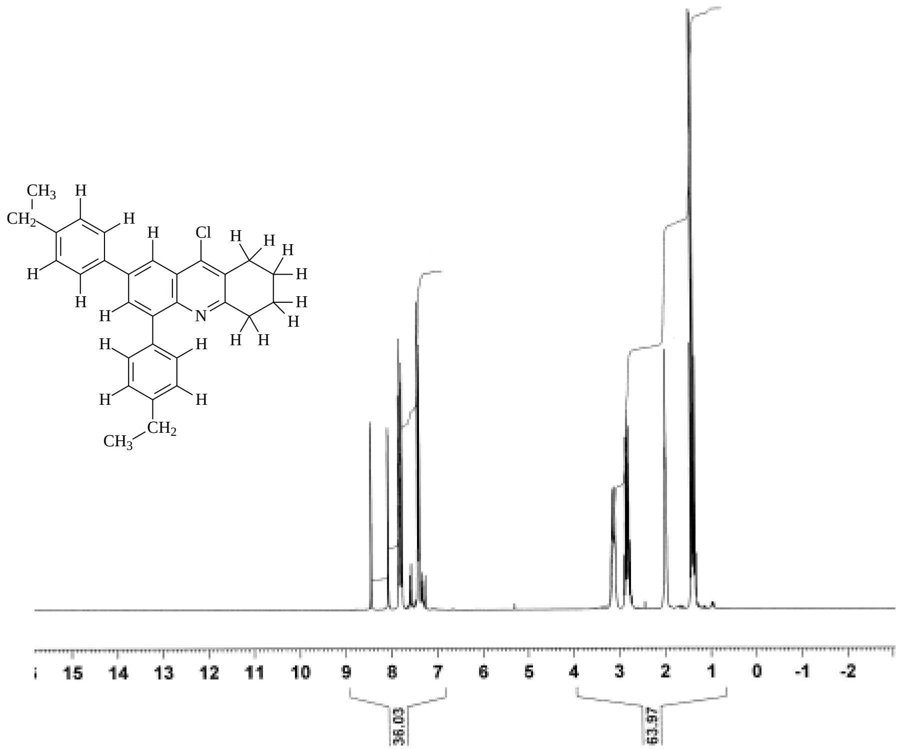

Figure S16: ^{1}H NMR (300 MHz, $CDCl_3$) of 2,4-bis(4-ethylphenyl)-9-chloro-5,6,7,8-tetrahydroacridine **(4e)**

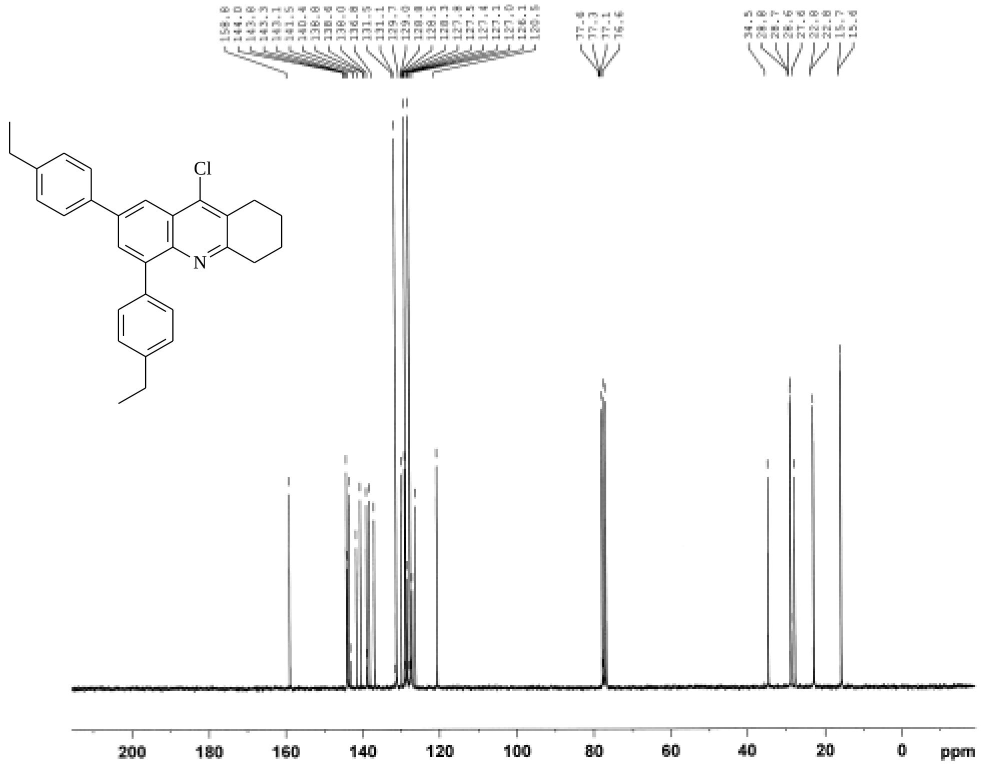

Figure S17: ^{13}C NMR (75 MHz, $CDCl_3$) of 2,4-bis(4-ethylphenyl)-9-chloro-5,6,7,8-tetrahydroacridine **(4e)**

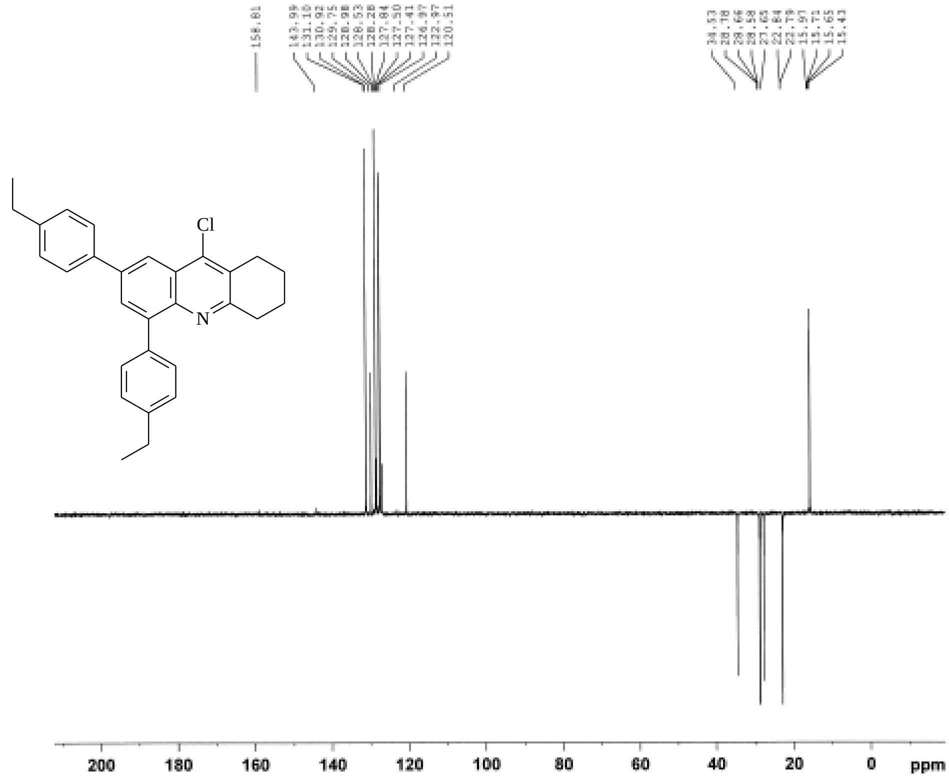

Figure S18: ^{13}C NMR DEPT 135 spectrum of 2,4-bis(4-ethylphenyl)-9-chloro-5,6,7,8-tetrahydroacridine **(4e)**

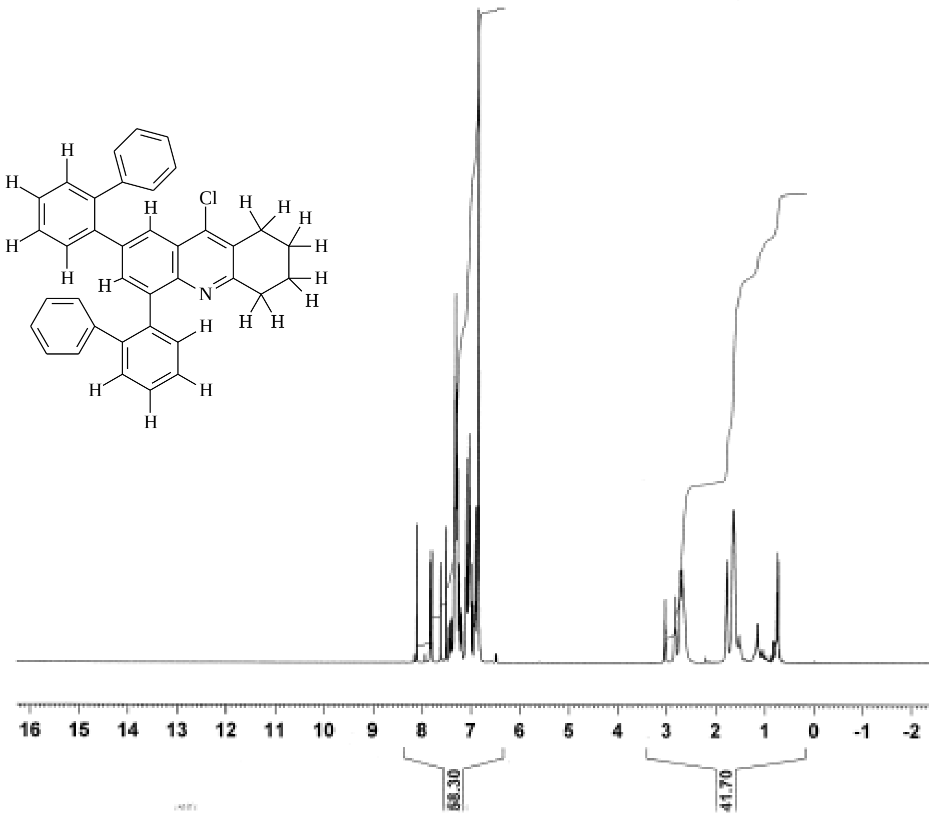

Figure S19: ^{1}H NMR (300 MHz, $CDCl_3$) of 2,4-bis(2-phenylphenyl)-9-chloro-5,6,7,8-tetrahydroacridine **(4f)**

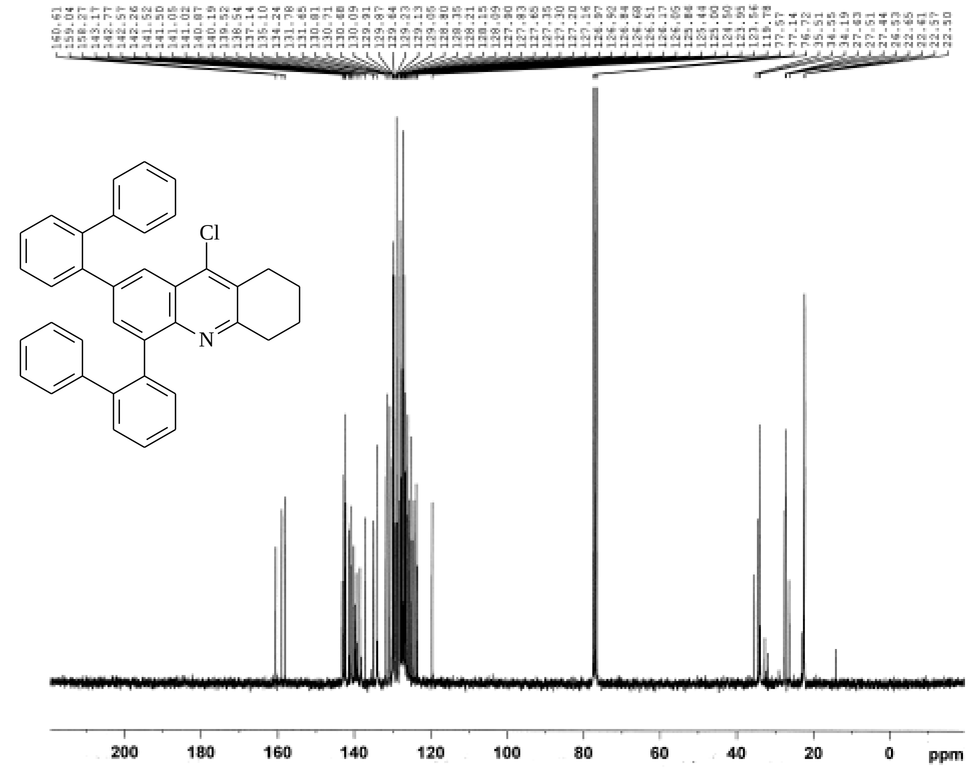

Figure S20: ^{13}C NMR (75 MHz, $CDCl_3$) of 2,4-bis(2-phenylphenyl)-9-chloro-5,6,7,8-tetrahydroacridine **(4f)**

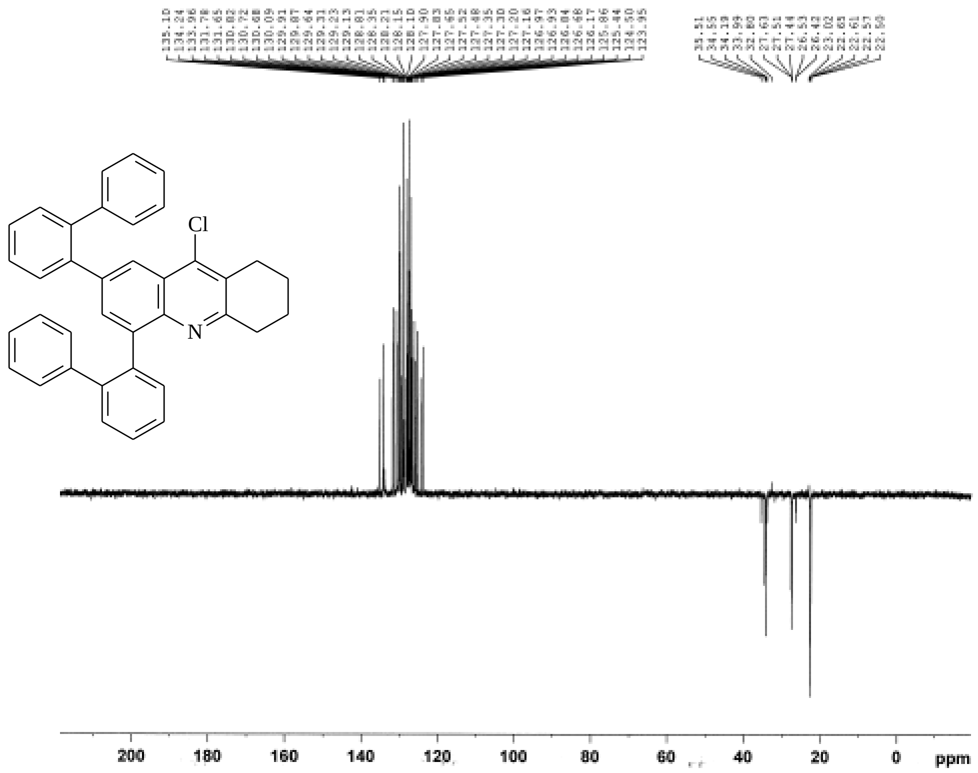

Figure S21: ^{13}C NMR DEPT 135 spectrum of 2,4-bis(2-phenylphenyl)-9-chloro-5,6,7,8-tetrahydroacridine **(4f)**
